# Brd/BET Proteins Influence the Genome-Wide Localization of the Kaposi’s Sarcoma-Associated Herpesvirus and Murine Gammaherpesvirus Major Latency Proteins

**DOI:** 10.3389/fmicb.2020.591778

**Published:** 2020-10-22

**Authors:** Rishikesh Lotke, Ulrike Schneeweiß, Marcel Pietrek, Thomas Günther, Adam Grundhoff, Magdalena Weidner-Glunde, Thomas F. Schulz

**Affiliations:** ^1^Institut für Virologie, Medizinische Hochschule Hannover, Hanover, Germany; ^2^German Center for Infection Research, Hannover-Braunschweig and Hamburg Sites, Hanover, Germany; ^3^Heinrich-Pette-Institut, Leibniz-Institut für Experimentelle Virologie, Hamburg, Germany

**Keywords:** KSHV, MHV-68, kLANA, mLANA, Brd/BET proteins, Brd2, Brd4, chromatin association

## Abstract

The *rhadinoviruses* Kaposi’s Sarcoma-associated herpesvirus (KSHV) and murine gammaherpesvirus (MHV-68) persist in infected hosts in a latent state that is characterized by the absence of virus production and by restricted viral gene expression. Their major latency protein, the latency-associated nuclear antigen (kLANA for KSHV and mLANA for MHV-68), is essential for viral genome maintenance and replication and involved in transcriptional regulation. Both kLANA and mLANA interact with cellular chromatin-associated proteins, among them the Bromodomain and Extra Terminal domain (Brd/BET) proteins, which recruit cellular and viral proteins to acetylated histones through their bromodomains and modulate cellular gene expression. Brd/BET proteins also play a role in the tethering, replication, segregation or integration of a diverse group of viral DNA genomes. In this study we explored if Brd/BET proteins influence the localization of the LANAs to preferential regions in the host chromatin and thereby contribute to kLANA- or mLANA-mediated transcriptional regulation. Using ChIP-Seq, we revealed a genome-wide co-enrichment of kLANA with Brd2/4 near cellular and viral transcriptional start sites (TSS). Treatment with I-BET151, an inhibitor of Brd/BET, displaced kLANA and Brd2/4 from TSS in the viral and host chromatin, but did not affect the direct binding of kLANA to kLANA-binding sites (LBS) in the KSHV latent origin of replication. Similarly, mLANA, but not a mLANA mutant deficient for binding to Brd2/4, also associated with cellular TSS. We compared the transcriptome of KSHV-infected with uninfected and kLANA-expressing human B cell lines, as well as a murine B cell line expressing mLANA or a Brd2/4-binding deficient mLANA mutant. We found that only a minority of cellular genes, whose TSS are occupied by kLANA or mLANA, is transcriptionally regulated by these latency proteins. Our findings extend previous reports on a preferential deposition of kLANA on cellular TSS and show that this characteristic chromatin association pattern is at least partially determined by the interaction of these viral latency proteins with members of the Brd/BET family of chromatin modulators.

## Introduction

Kaposi’s Sarcoma-associated herpesvirus (KSHV), the etiological agent of Kaposi’s sarcoma (KS) (Chang et al., 1994), is also responsible for two lymphoproliferative diseases, Multicentric Castleman’s Disease (MCD), and primary effusion lymphoma (PEL) (Chang et al., 1994; Cesarman et al., 1995; Soulier et al., 1995) al., 1994; Soulier et al., 1995) and a rare plasmablastic lymphoma arising in MCD lesions (Dupin et al., 2000). Like other herpesviruses, it establishes a life-long latent infection, which is characterized by a restricted viral gene expression pattern in infected cells. Of the few viral genes expressed in the latent phase, latency-associated nuclear antigen (LANA) is essential for the establishment and maintenance of latency. It is a multifunctional protein that is important for viral genome maintenance in dividing cells through viral genome replication, tethering to host mitotic chromosomes and segregation, and is involved in transcriptional regulation of both viral and cellular genes. LANA executes its functions by associating with chromatin directly or indirectly through its N- or C-terminal chromatin binding domains. The N-terminal LANA domain binds directly to histones H2A/B, H2AX, H3, and H1 or indirectly through Methyl-CpG-binding protein 2 (MeCP2) (Cotter and Robertson, 1999; Krithivas et al., 2002; Shinohara et al., 2002; Barbera et al., 2004, 2006a, b; Lim et al., 2004; Jha et al., 2013; Kim et al., 2013; Verma et al., 2013). The C-terminal domain binds directly to its cognate LANA-binding sites (LBS) LBS1, LBS2, and LBS3 within the terminal repeat (TR) of the viral genome and also associates indirectly with chromatin through chromatin-associated proteins MeCP2 and Brd/BET proteins Brd2 and Brd4 (Platt et al., 1999; Garber et al., 2002; Mattsson et al., 2002; Viejo-Borbolla et al., 2003, 2005; Ottinger et al., 2006; You et al., 2006; Griffiths and Whitehouse, 2007; Kelley-Clarke et al., 2007; Matsumura et al., 2010; Domsic et al., 2013; Hellert et al., 2013, 2015) with the cellular chromatin in a genome-wide manner have shown that it is preferentially deposited in the vicinity of cellular transcription start sites (TSS) (Lu et al., 2012; Hu et al., 2014; Mercier et al., 2014; Lin et al., 2017). However, the reasons for this observed preferential association and its associated functional implications are not yet fully understood.

Members of the Brd/BET protein family contain two bromodomains and one extraterminal (ET) domain (Florence and Faller, 2001; Zeng and Zhou, 2002). Bromodomains recognize particular protein acetylation patterns, mainly but not exclusively on histones, while the ET domain is involved in protein-protein interactions with other cellular proteins (Dhalluin et al., 1999; Platt et al., 1999; Denis, 2001; Yang et al., 2005; Denis et al., 2006; Peng et al., 2007; Rahman et al., 2011; LeRoy et al., 2012; Anders et al., 2014; Fujisawa and Filippakopoulos, 2017). Brd/BET family members therefore act as ‘readers’ of histone acetylation and direct other cellular proteins to particular histone acetylation patterns. ChIP-Seq studies indicate that Brd2 and Brd4 are preferentially located in the vicinity of cellular TSS (LeRoy et al., 2012; Anders et al., 2014). Among the five human Brd/BET family members, Brd2 has been shown to be involved in transcriptional regulation of cellular genes and it upregulates cell cycle genes such as cyclin D1, cyclin A, cyclin E, and dihydrofolate reductase by interaction with the E2F transcription factor (Denis and Green, 1996; Denis et al., 2000). Brd2 acts as a scaffold as it mediates E2F recruitment and assembly of RNA PolII, TATA binding protein (TBP), TBP associated factors (TAF) and nucleosome remodeller, SWI/SNF complex, by associating with the RNA PolII mediator complex (Denis, 2001; Denis et al., 2006; Peng et al., 2007). Brd4 also has been shown to be involved in the regulation of cellular and viral genes. Similar to Brd2, it acts as a scaffold at promoters and enhancers (Denis, 2001; Fujisawa and Filippakopoulos, 2017) for recruiting transcription factors and the active form of positive transcription elongation factor b (P-TEFb), which associates with the PolII mediator complex (Wu and Chiang, 2007), thereby promoting transcriptional elongation by PolII (Jang et al., 2005; Yang et al., 2005). In addition to histone acetylation recognition, Brd4 has also been shown to have histone acetyl transferase (HAT) activity by acetylating lysine K122 of histone H3. This is important for nucleosome stability and it promotes chromatin decompaction resulting in enhanced transcription (Devaiah et al., 2016).

Several studies have shown the involvement of the Brd/BET proteins in the life cycle of different viruses. Brd4 interacts with the large T antigen (LT) of Merkel cell polyomavirus (MCV) and recruits cellular DNA replication factors for MCV DNA replication (Wang et al., 2012). Brd4 is recruited by E2 of Human Papillomavirus 16 (HPV16) at the foci of viral replication and can stimulate the replication. It also mediates the tethering of the Bovine Papillomavirus (BPV) genome to mitotic chromosomes (You et al., 2004; Wu et al., 2006; Olejnik-Schmidt et al., 2008; Sakakibara et al., 2013; Jang et al., 2014). In the case of Epstein Barr Virus (EBV), it has been shown that Brd4 enables EBNA1-mediated segregation of the viral genome (A. Lin et al., 2008) and plays a role in the replication of the EBV genome by binding to its lytic origin of replication (Keck et al., 2017). Brd/BET proteins also interact with the integrase of γ-retroviruses Murine leukemia virus (MLV) and Feline Leukemia virus (FeLV) and are responsible for targeting the viral pre-integration complex into the proximity of TSS of cellular genes and CpG islands, thereby determining the classical γ-retroviral integration pattern in the vicinity of cellular TSS (De Rijck et al., 2013; Gupta et al., 2013; Sharma et al., 2013). Additionally, the *piggyBac* (PB) transposase was shown to interact with Brd4 and the integration pattern of the PB transposon was found to be similar to that of MLV and to be Brd4-dependent (Gogol-Doring et al., 2016).

In the case of the two γ2 herpesviruses (*Rhadinoviruses*) KSHV and MHV-68, Brd/BET proteins have been shown to interact with KSHV LANA (kLANA) and its MHV-68 homolog mLANA and to play a role in transcriptional regulation, chromatin association and latent episomal replication (Platt et al., 1999; Mattsson et al., 2002; Viejo-Borbolla et al., 2003, 2005; Ottinger et al., 2006, 2009; You et al., 2006; Hellert et al., 2013). In view of the notion that Brd/BET proteins direct cellular and viral proteins to chromatin regions displaying particular histone acetylation patterns and thereby, as summarized above, the association of some viral genomes with a specific chromatin environment, we explored if they might determine the preferential association of kLANA and mLANA with cellular TSS.

Our findings indicate that the Brd/BET proteins, Brd2 and Brd4, influence the association of LANA with the TSS of actively transcribed cellular genes.

## Materials and Methods

### Cell Culture, Reagents, and Treatment Conditions

The establishment of BJAB cells expressing mCherry or kLANA, and of the murine B cell line A20 expressing GFP (A20-GFP), MHV-68 LANA (A20-mLANA WT) or its mutant (A20-mLANA 3A) is described below. The establishment of BJAB-rKSHV.219 cells has been previously described (Kati et al., 2013, 2015). BCBL-1, BJAB, BJAB-mCherry, BJAB-kLANA and BJAB-rKSHV.219 were cultured in RPMI 1640 (Gibco) with 20% (v/v) Foetal Calf Serum (FCS) (HyClone), whereas A20-GFP, A20-mLANA WT and A20-mLANA 3A mutant were cultured in RPMI 1640 with 10% (v/v) FCS. HEK293T were cultured in DMEM (Gibco) with 10% (v/v) FCS. All cells were incubated in a humidified incubator with 5% CO_2_ at 37°C. The inhibitor I-BET151 (GSK1210151A) was purchased from Chemietek. BCBL-1, BJAB-mCherry, BJAB-kLANA and BJAB-rKSHV.219 cells were plated in T-75 flasks at a cell density of 0.5 × 10^6^ cells/ml and in the absence or presence of I-BET151 at a final concentration of 0.5 μM for 48 h. Lytic cycle inducer sodium butyrate (SB) was used at a final concentration of 1.25 mM along with 5% (v/v) of supernatant of Sf9 cells infected with a recombinant baculovirus expressing the KSHV replication and transcription activator (RTA). 12-O-tetradecanoylphorbol-13-acetate (TPA) was used at a final concentration of 50 ng/ml. For induction of A20-GFP, A20-mLANA WT or A20-mLANA 3A, cells were cultured in a T-75 flask at a density of 0.2 × 10^6^ cells/ml supplemented with doxycycline at a final concentration of 1 μg/ml.

### Construction of kLANA and mLANA Lentiviral Vectors, Lentivirus Production and Generation of kLANA, mLANA WT, and mLANA 3A Mutant Expressing Stable Cell Lines

Full length kLANA was excised from pGTR4:73 (Grundhoff and Ganem, 2003) using EcoRI and BclI and cloned into the pSL301 (Invitrogen^TM^) vector to yield pSL301-kLANA. Next, the pSRS11_SF mCherry PRE mCMV EGFP vector (gift from Jens Bohne, Medizinische Hochschule Hannover) was digested using AgeI and SgrAI to excise the region coding for mCherry and the resulting vector backbone was Klenow-filled and self-ligated. Finally, the intermediate pSL301-kLANA vector was digested with NotI to release a fragment containing full-length kLANA, which was then cloned into the NotI site of the Klenow-filled lentiviral backbone to generate the kLANA expressing lentiviral vector pSRS11_SF kLANA PRE mCMV EGFP.

The construction of the mLANA lentiviral vectors was as follows. The lentiviral vector pSERS11 lmg (gift from Christopher Baum, Medizinische Hochschule Hannover) contains an M2 dependent T6 (doxycycline-inducible minimal CMV) promotor for mRNA expression. This vector was digested using NotI and MluI and the 4957 bp fragment was gel eluted. Another restriction digestion on pSERS11 lmg was carried out in parallel using MluI and SalI and the 1506 bp fragment was gel eluted. The IRES-EGFP region in the pSF91 retroviral vector (gift from Christopher Baum, Medizinische Hochschule Hannover) was PCR amplified using the primers SalI-IRES-EGFP fwd (5′-TATGTCGACGCCACCATGGTGAGCAAGGGCGAGGAG-3′) and IRES-EGFP-NotI rev (5′-TATGCGGCCGCTTACTTGTAC AGCTCGTCCATGCC-3′). The 734 bp amplicon containing a SalI restriction site at its 5′ end and a NotI site at its 3′ end was digested using SalI and NotI. This digested amplicon, the initially obtained 4957 bp and 1506 bp fragments were ligated together to yield the pSERS IRES-EGFP vector (GFP-Control vector). A pSERS MHV68-ORF73 FLAG vector was obtained in a similar manner. However, the 983 bp MHV68-ORF73 FLAG insert was obtained by performing restriction digestion using SalI and NotI on a codon optimized, MHV-68-ORF73 FLAG containing vector from Gene Art Ltd. Regensburg, Germany. Next, the pSERS MHV68-ORF73 FLAG vector was linearized using NotI and then treated with Alkaline Phosphatase, Calf Intestinal (CIP). The IRES-EGFP region in the pSF91 vector was amplified using the primers NotI-IRES-EGFP fwd (5′-TATGCGGCCGCCCCCTCTCCCTCCCCCCC-3′) and IRES-EGFP-NotI rev to yield an amplicon with a NotI restriction site at both the 5′ and 3′ ends. This amplicon was digested using NotI and was ligated to the linearized pSERS MHV68-ORF73 FLAG vector to yield the pSERS MHV68-ORF73 FLAG-IRES-EGFP vector (mLANA WT). The correct orientation of the insert was checked by restriction digestion using HindIII. Finally, the pSERS MHV68-ORF73 3A FLAG-IRES-EGFP vector (mLANA 3A) was generated by site directed mutagenesis of pSERS MHV68-ORF73 FLAG-IRES-EGFP using the two primer pairs K224A, K228A fwd (5′-CT TTCATTTGTTGAAGACGCGAAACAGGCCGCAAAACTAAA AAGG-3′) – K224A, K228A rev (5′-CCTTTTTAGTTT TGCGGCCTGTTTCGCGTCTTCAACAAATGAAAG-3′) and K169A fwd (5′-CACCCCCCAACACATTTTGCGTCAGCTGTT ATGTTTAGTAGC-3′) – K169A rev (5′-GCTACTAAACATA ACAGCTGACGCAAAATGTGTTGGGGGGTG-3′).

Lentiviruses were produced using expression vectors for M57 DAW (lentiviral gag/pol), RD114 envelope protein (both a gift from Jens Bohne, Medizinische Hochschule Hannover) and the constructed lentiviral vectors as described previously in (Zhang et al., 2016).

To generate a stable kLANA-expressing BJAB cell line, 2 × 10^6^ BJAB cells were transduced with the lentivirus coding for kLANA. Cells were sorted using FACS for EGFP positive cells and further cultured. A second round of FACS sorting was carried out and cells were cryopreserved after two passages of the enriched population. Cells were sorted twice to a minimum of 95% purity.

To generate the mLANA-expressing A20 cell line, 2 × 10^5^ A20 cells were resuspended in 1 ml of complete medium [RPMI 1640 with 10% (v/v) FCS] and mixed along with the lentivirus and 4 μg/ml of protaminsulfate per well of a 12-well plate. The plate was centrifuged at 450 × *g* for 30 min at 30°C and incubated at 37°C for 6 h in a humidified CO_2_ incubator. The cells were washed and resuspended into complete medium. Cells were grown and passaged twice before inducing with doxycycline at a final concentration of 1 μg/ml for 48 h and sorting them by FACS for EGFP positive cells. A20-GFP control cells were established using the pSERS IRES-EGFP lentivirus, the A20-mLANA WT using the pSERS MHV68-ORF73 FLAG-IRES-EGFP lentivirus and finally the A20-mLANA 3A using the pSERS MHV68-ORF73 3A FLAG-IRES-EGFP lentivirus. Cells were sorted twice to a minimum of 95% purity.

### Immunofluorescence

The staining was performed as described previously (Hellert et al., 2013) with following changes: cells were washed and resuspended in PBS to a concentration of 0.5 × 10^6^ cells/ml to 1 × 10^6^ cells/ml and 10–15 μl of the cell suspension was dropped and allowed to dry on Superfrost/Plus slides (Thermo Fisher Scientific). A circle surrounding the dried cells was drawn with a hydrophobic pen (PAP PEN). The fixation with 4% PFA, quenching, permeabilization and blocking was performed as in Hellert et al. (2013). Incubation with the primary antibodies (α-ORF59, Advanced Biotechnologies Inc. #13-211-100, dilution 1:100; α-LANA, NovocastraLiquid^TM^ Mouse Monoclonal α-LANA NCL-L-HHV8-LNA, dilution 1:100), secondary antibodies (Jackson ImmunoResearch Laboratories, Inc., Cy^TM^ 3-labeled Donkey Anti-Mouse IgG #715-165-150, dilution 1:200; and Cy^TM^ 5-conjugated AffiniPure Goat Anti-Mouse IgG #115-175-146, dilution 1:200) and washes were performed in 10% FCS in PBS + 0.2% Triton X-100. Slides were then washed with PBS, stained with DAPI (1 mg/ml, 1:1000) for 30 min at 37°C and mounted with MOVIOL containing DABCO (2.5%). Slides were dried overnight at room temperature in the dark and imaged the next day using the Zeiss Z1 AxioObserver using the Plan-Neofluar 20×/0.50 objective (ORF59) or the Plan-Apochromat 63×/1.40 Oil DIC M27 objective (kLANA).

### Chromatin Immunoprecipitation (ChIP)

Chromatin Immunoprecipitation was performed as described in Si et al. (2006), Gunther and Grundhoff (2010) with the following modifications. For kLANA and mLANA ChIP, the formaldehyde fixation time was 10 min, while for Brd2/4 it was 20 min. For the Brd2/4 ChIP experiments we used rabbit polyclonal antigen-affinity purified IgG antibodies (Bethyl laboratories, Inc. BRD2-A302-583A and BRD4-A301-958A100) at a concentration of 3 μg/mg of lysate. Antibodies were incubated with the lysates for 6 h before adding protein G beads and then further incubated for 16 h. For kLANA ChIP, we used an IgG2c rat monoclonal antibody (Advanced Biotechnologies Inc. 13-210-100) and prepared antibody-conjugated beads as previously described (Zhang et al., 2016), with the exception of the beads being washed and finally resuspended in ChIP dilution buffer (0.01% SDS, 1.1% Triton X-100, 1.2 mM EDTA, 16.7 mM Tris HCl – pH 8.0, 167 mM NaCl). Rabbit IgG (Cell Signaling Technology^®^ #2729) and Rat IgG (Abcam ab37361) were used as isotype controls in, respectively, the Brd2/4 and kLANA ChIP experiments. For the mLANA experiments, we used 50 μl of EZview^TM^ Red anti-FLAG^®^ M2 Affinity Gel (Sigma-Aldrich) per lysate derived from ∼3 × 10^7^ nuclei.

ChIP-qPCR was performed using the DyNAmo HS SYBR Green qPCR Kit from Thermo Fisher Scientific according to the manufacturer’s instructions on a Stratagene Mx3000P qPCR instrument from Agilent technologies or an ABI 7500 real-time PCR instrument (Applied Biosystems). The primer pairs used were as follows: KSHV TR (Cheng et al., 2011) 5′- TGTGTGTGAGCCTGTTTG-3′ and 5′-TGTTCACGTAGTGTCCAG-3′; Human TRIM2 5′-AG TCGCCGTCTCGTAGTAAGTC-3′ and 5′- CTCTGGGCAG GCTTCCCG-3′; Human IQGAP3 5′-TTGTAGTTCCCGG CCGCGGAG-3′ and 5′- ACAAGACGAGGAACTGCTGTAAG GG-3′; Mouse Rcan3 5′- GAGAGGCCCGAGGGGGGATGC-3′ and 5′- TGAGGCGGCCGCTGCGG-3′; Mouse Tm2D1 5′- GG GAAGCCCGACCCGCGG-3′ and 5′- CCAGGCCCCTTTA AGGCCCTTCC-3′.

ChIP-Seq libraries were prepared using the NEXTflex^TM^ ChIP-Seq and NEXTflex^TM^ ChIP-Seq Barcodes – 12 kits from BIOO Scientific Corp. according to the manufacturer’s instructions (NEXTflex^TM^ ChIP-Seq kit Manual 5143-02) to prepare single-end DNA libraries from ChIP DNA using the gel-free protocol and libraries were size selected to be between 300 and 400 bp. The NEXTflex^TM^ ChIP-Seq Barcodes were used during the adapter ligation step. ChIP DNA and ChIP-Seq libraries were quantified using the Qubit^®^ dsDNA HS Assay Kit as per the manufacturer’s instructions and library size distribution determined using the Agilent 2100 Bioanalyzer chip according to manufacturer’s instructions. The ChIP-Seq libraries were sequenced using the Illumina^®^ TruSeq SBS v3-HS (50 cycles) and Illumina^®^ NextSeq 500/550 High Output v2 (75 cycles) kits according to the manufacturer’s instructions on the Illumina^®^ HiSeq^®^ 2500 and Next-Seq^®^ platforms, respectively. Cluster generation for the HiSeq^®^ was performed using the TruSeq SR Cluster Kit v3-cBot-HS on an Illumina^®^ cBOT^TM^ machine.

### ChIP-Seq Analysis

#### QC and Pre-processing

All FASTQ sequence files were checked for quality using the FastQC tool. Reads exceeding 51 bp were trimmed from the 3′ end down to 51 bp using the FASTQ/A trimmer tool from the FASTX-toolkit.

#### Read Alignment

We used Bowtie for aligning reads to the mouse (mm9) genome for mouse datasets, and human (hg19) genome for human datasets, using the default options with the following exceptions: –best, –strata, and -m 1. For data from cells containing the KSHV genome, reads were also aligned to the HQ404500 sequence to which two copies of TRs were appended. Alignment of the reads to the long unique region (LUR) was performed with the aforementioned settings. All resulting SAM files were sorted, duplicates were removed and finally converted to BAM format.

#### Peak Calling

Peak calling was performed using MACS using the default options with the following exceptions: –nomodel and –shiftsize value was set to half the size of mean fragment length of the ChIP-Seq library as determined by Agilent 2100 Bioanalyzer. For KSHV LUR, peak calling was performed with the aforementioned settings but with the genome size set to 137706 bp and using an additional option –nolambda. For alignment to the TR regions of the KSHV genome, reads were aligned using Bowtie with the aforementioned options for read alignment, except -m 1 was changed to -M 1. The SAM files were sorted and converted to BAM without removal of duplicates. No peak calling was performed for alignments for the TR. Instead, the aligned reads were extended to match the library fragment size using the igvtools ‘Count’ command from the Integrative Genomics Viewer (v2.3) with default settings, the window size set to 10 and the extension factor set depending on the average fragment size of the library (Langmead et al., 2009; Langmead, 2010; H. Li et al., 2009; Robinson et al., 2011; Thorvaldsdottir et al., 2013). We used the Bedtools toolset to identify concordant enriched regions amongst biological replicates using the *intersect* sub-tool and the *closest* subtool for annotating peaks to the closest gene (Quinlan and Hall, 2010). Venn Diagrams were prepared using Microsoft PowerPoint (v14.0.7192.5000. 32-bit). To measure the average ChIP enrichment signals over a 3 kbp meta-gene and in a region of ±3 kbp from the TSS of genes, we used the Cis-regulatory Element Annotation System (CEAS) (Ji et al., 2006; Shin et al., 2009) with the default options and with –pf-res = 10. Average fold enrichment signals over a 3 kbp meta-gene and in a region of ±3 kbp from the TSS of genes were normalized with respect to the input signal based on a scaling factor derived from the total number of reads.

#### Motif Analysis

Motif analysis was performed using the online MEME-ChIP tool from the Multiple Em for Motif Elicitation (MEME) suite. 50 bp were added upstream and downstream of the peak summits coordinates in order to get coordinates for a sequence length of 100 bp. Fasta sequences for these coordinates were then obtained using the ‘Extract Genomic DNA’ tool from the Galaxy web-based platform using the appropriate genome as reference. The obtained fasta file was used as input for the MEME-ChIP program, which was run using the default parameters against the HOCOMOCO COmprehensive MOdel COllection (HOCOMOCO v10) database for human DNA motifs. Motifs and Centrimo results were obtained as standard outputs post-analysis. For the Find Individual Motif Occurrences (FIMO) scan analysis, KSHV LBS1 motif CCGCCCGGGCATGGGG was provided as input, the database selected was UCSC Mammal genomes, the organism selected was human and genome version hg19 (Grant et al., 2011; Machanick and Bailey, 2011; Kulakovskiy et al., 2013; Afgan et al., 2016).

#### ChromHMM

To identify the chromatin states associated with peak regions from our ChIP-Seq data, we used the ChromHMM tool with the default settings and set a bin size of 200 bp and 13 states. BAM files for the human epigenetic factors were extracted from the UCSC genome browser downloads portal for the GM12878 cells from the ENCODE/Broad Institute datasets. BAM file for all replicates were merged using the SAM tools merge feature to obtain a single merged BAM file for each epigenetic mark (Kent et al., 2002; Consortium, 2012; Ernst and Kellis, 2012).

### Microarray

Total RNA from BJAB-mCherry, BJAB-kLANA and BJAB-rKSHV.219 cells in the absence and presence of 0.5 μM I-BET151 for 48 h and from A20-GFP, A20-mLANA WT and A20-mLANA 3A cells in the absence and presence of 1 μg/ml doxycycline for 24 and 48 h was isolated using the Qiagen RNeasy^®^ Mini kit. For the human samples, the Microarray utilized in this study represents a refined version of the Whole Human Genome Oligo Microarray 4 × 44K v2 (Design ID 026652, Agilent Technologies), called ‘026652QM_RCUG_HomoSapiens’ (Design ID 084555) developed by the Research Core Unit Genomics (RCUG) of the Medizinische Hochschule Hannover. Microarray design was created at Agilent’s eArray portal using a 1 × 1M design format for mRNA expression as template. All non-control probes of design ID 026652 were printed five times within a region comprising a total of 181560 Features (170 columns × 1068 rows). Four of such regions were placed within one 1M region giving rise to four microarray fields per slide to be hybridized individually (Customer Specified Feature Layout). Control probes required for proper Feature Extraction software operation were determined and placed automatically by eArray using recommended default settings. 375 ng of total RNA was used for synthesis of aminoallyl-UTP-modified (aaUTP) cRNA with the ‘Quick Amp Labeling kit, no dye’ (#5190-0447, Agilent Technologies) according to the manufacturer’s recommendations, except that reaction volumes were quartered and the NTP-mix contained in the kit was exchanged for NTP Set (ATP, CTP, GTP, and UTP) and aminoallyl-UTP (Fermentas, Thermo Fisher Scientific; order numbers R1091, R0481, respectively). Final NTP concentrations used for in-vitro transcription were 2.5 mM (ATP, CTP, and GTP) and 1.25 mM (UTP, aaUTP). The labeling of aaUTP-cRNA was performed by use of Alexa Fluor 555 Reactive Dye (#A32756; Life Technologies) as described in the Amino Allyl Message Amp^TM^ II Kit Manual (#AM1753; Life Technologies) except that reaction volumes were quartered. Prior to the reverse transcription reaction, 0.75 μl of a 1:1000 dilution of Agilent’s ‘One-Color spike-in Kit stock solution’ (#5188–5282, Agilent Technologies) were added to each 375 ng of total RNA sample. For the mouse samples, The Whole Mouse Genome Oligo Microarray Kit 4 × 44 v2 (G4846A, design ID 026655, Agilent Technologies) was utilized in this study. Microarrays of this design type contain 44397 oligonucleotide probes covering roughly 30000 murine transcripts. Synthesis of the Cy3- or Cy5-labeled cRNA was performed with the ‘Quick Amp Labeling kit, two color’ (#5190-0444, Agilent Technologies) according to the manufacturer’s recommendations. For the human samples, cRNA fragmentation, hybridization and washing steps were carried out as recommended in the ‘One-Color Microarray-Based Gene Expression Analysis Protocol V5.7,’ except that 1000 ng of each fluorescently labeled cRNA population were used for hybridization. For the mouse samples cRNA fragmentation, hybridization and washing steps were carried out as recommended in the ‘Two-Color Microarray-Based Gene Expression Analysis Protocol V5.7,’ except that 2000 ng of each labeled cRNA population were used for hybridization. Slides were scanned on the Agilent Micro Array Scanner G2565CA (pixel resolution 3 μm for the human array and 5 μm for the mouse array, bit depth 20). Data extraction was performed with the ‘Feature Extraction Software V10.7.3.1’ using the extraction protocol file ‘GE1_107_Sep09.xml’ for the human data and ‘GE2_107_Sep09.xml’ for the mouse data. Measurements of on-chip replicates (quintuplicates in the case of human array) were averaged using the geometric mean of processed intensity values of the green channel, ‘gProcessedSignal’ (gPS) to generate one single value per unique non-control probe, whereas for the mouse array, replicates were averaged using the geometric mean of ProcessedSignal (PS) values (for each channel independently) to generate one single value per probe, sample, and channel. Individual signals were excluded from averaging, if they (i) were manually flagged, (ii) were identified as Outliers by the Feature Extraction Software, (iii) lay outside the interval of ‘1.42 × interquartile range’ regarding the normalized gPS distribution of the respective on-chip replicate population, or, (iv) showed a coefficient of variation of pixel intensities per signal that exceeded 0.5. For the human arrays, averaged gPS values were normalized by global linear scaling: For human arrays, all gPS values of one sample were multiplied by an array-specific scaling factor and for the mouse arrays, all PS values (gPS and rPS) of one particular microarray were multiplied with a microarray-specific scaling factor. The scaling factor for the human arrays was calculated by dividing a ‘reference 75th Percentile value’ (set as 1500 for the whole series) by the 75th Percentile value of the particular Microarray to be normalized (‘Array I’ in the formula shown below) and for the mouse arrays by the 75th Percentile value of the gPS calculated by the Feature Extraction software for that microarray (‘Array i’). Accordingly, normalized gPS values for all samples (microarray data sets) were calculated by the following formula:

$$\begin{array}{rcl} normalized\;gPS_{Array\;I} & = & gPS_{Array\;I}\times \\ & & \left(1500/75th\;Percentile_{Array\;I}\right)human\;data \end{array}$$

$$\begin{array}{rcl} normalized\;PS_{Array\;i} & = & PS_{Array\;i}\times \\ & & (1500/75th\;Percentile\;gPS_{Array\;i})mouse\;data \end{array}$$

Finally, a lower intensity threshold (surrogate value) was defined based on intensity distribution of negative control values. This surrogate value was fixed at 10 normalized gPS units for human arrays and 15 normalized PS units for mouse arrays. All measurements that fell below this intensity cutoff were substituted by the respective surrogate value. For analysis, ratio values of relative gene expression were calculated using Microsoft Excel macros. The intensity cut-off was set to 50. Fold change was calculated by averaging over the three biological replicates then taking ratios and the resulting values were log_2_ transformed. The fold change cut-off was set to 1.5 fold. A two-tailed paired *t*-test, was performed on log_2_ transformed normalized intensity values. A *p*-value ≤ 0.05 was considered significant. Volcano plots were prepared by plotting -log_10_ transformed *p*-values against the log_2_ transformed fold change values. Upregulated probes/genes were indicated as red dots, downregulated were green and those not differentially regulated were gray. For box plots log_2_ transformed normalized processed signal values were plotted. A Mann-Whitney test was performed for box plots to check for significance between the median values.

### Genomics Data Access

The data discussed in this publication have been deposited in NCBI’s Gene Expression Omnibus (GEO) and are accessible through GEO SuperSeries accession number GSE153244^1^.

### Statistical Analysis

Statistical analysis was performed using GraphPad Prism 5 (v5.02). One-way ANOVA was performed for quantification of ORF59-expressing cells, two-tailed unpaired *t*-tests for ChIP-qPCRs and Mann-Whitney *U* test for difference of medians and gene expression distributions.

## Results

### The Association of kLANA With the Long Unique Region (LUR) of the Viral Genome Involves Brd/BET Proteins

As reviewed in the introduction, kLANA is known to bind directly to three LANA binding sites (LBS) present in each TR subunit of the viral genome (Garber et al., 2002; Kelley-Clarke et al., 2007; Hellert et al., 2015) and was also shown to be associated with regions in the LUR of the KSHV genome by ChIP-Seq (Lu et al., 2012; Hu et al., 2014; Mercier et al., 2014; Sun et al., 2017). ChIP-Seq results revealed that kLANA is deposited in regions that carry histone modifications H3ac, H3K9/14ac and H3K4me3, typical of transcriptionally active chromatin. Deposition of kLANA on the LUR is negatively correlated with the constitutive heterochromatic mark H3K9me3 (Gunther and Grundhoff, 2010; Toth et al., 2010, 2013, 2016; Hu et al., 2014; Sun et al., 2017). In addition, kLANA has been shown to interact with the ET domain of the Brd/BET proteins Brd2 and Brd4 (Platt et al., 1999; Viejo-Borbolla et al., 2005; Ottinger et al., 2006; Hellert et al., 2013), which bind to acetylated histones H2AK5ac, H2AK36ac, H2AK75ac, H2BK43ac, H3ac, H3K18ac, H3K36ac, H3K37ac, H3K56ac, H4ac, H4K5ac, H4K5/8ac, H4K12ac, H4K20ac, H4K44ac, H1K74ac, and are therefore also preferentially found in open chromatin regions (Marushige, 1976; Dey et al., 2003; Kanno et al., 2004; Nakamura et al., 2007; Vollmuth et al., 2009; Umehara et al., 2010a, b; Philpott et al., 2011; Filippakopoulos et al., 2012).

**FIGURE 1:**
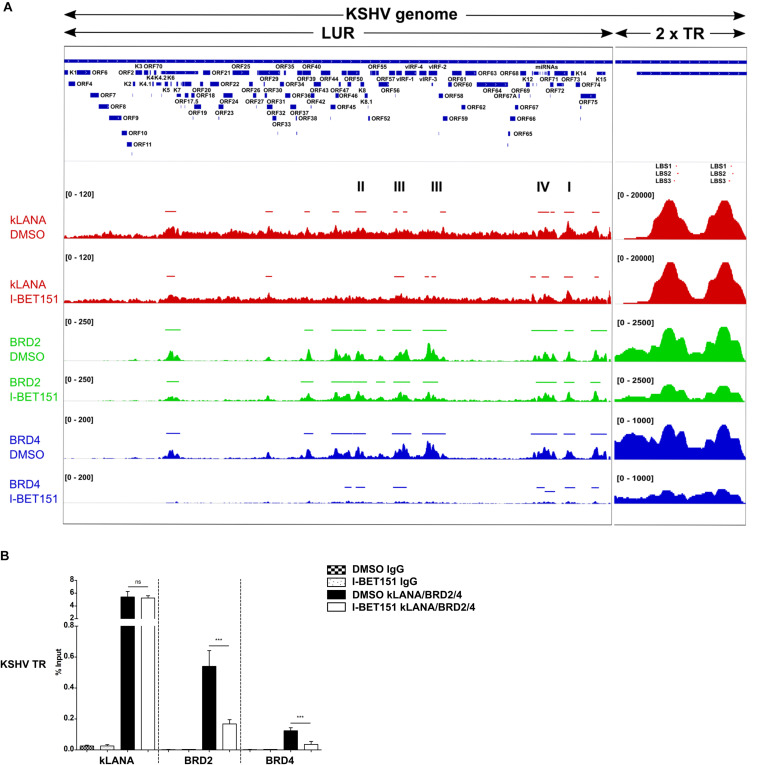
Association of kLANA, Brd2 and Brd4 with the viral genome. **(A)** ChIP-Seq for kLANA, Brd2 and Brd4 was performed in BCBL-1 cells treated or not with 0.5 μM I-BET151 for 48 h. Sequencing reads obtained were aligned to the KSHV genome (HQ404500 sequence) appended with two copies of the TR. **Top:** Diagram of the KSHV genome. **Bottom:** Deposition of kLANA, Brd2 and Brd4 on the KSHV genome in the presence or absence (DMSO) of I-BET151. Horizontal colored bars above the ChIP-Seq diagrams indicate peaks of kLANA, Brd2, and Brd4 deposition. I, latency control region; II, lytic control region; III, vIRF3; IV, miRNA region. The values in square brackets represent the number of extended reads. **(B)** ChIP-qPCR for kLANA, Brd2 and Brd4 in the presence or absence of I-BET151. PCR primer pair used here target the terminal repeat (TR) subunit near LBS1-3. Bars represent mean ± SD. ***p < 0.001.

In order to explore if Brd2 or Brd4 might play a role in the association of kLANA with the KSHV LUR, we performed ChIP-Seq experiments for kLANA, Brd2, and Brd4 in the PEL cell line BCBL-1 in the absence or presence of 0.5 μM I-BET151, a small molecule inhibitor that prevents Brd2 and Brd4 from binding to acetylated histones by occupying predominantly the first bromodomain of Brd2 and Brd4 (Chung et al., 2011; Dawson et al., 2011). Combining datasets from two independent experiments, we found that kLANA, Brd2, and Brd4 occupied several regions on the KSHV genome and that their enrichment was highest at LBS-1, 2 and 3 in the TR. After taking into account an assumed number of 40 TR units, peak heights for kLANA were approximately 200 fold greater at the LBS in the TR region compared to the LUR region. For Brd2 and Brd4, peak heights were approximately 5–10 fold higher at the LBS compared to the LUR region. All three proteins were, however, moderately enriched at several loci in the LUR, in particular the lytic and latency control regions, the vIRF and microRNA loci (Figure 1A). kLANA showed a broadly distributed enrichment pattern across a large section of the KSHV LUR, in which 12 kLANA peaks, significantly enriched above background (marked with horizontal lines above the kLANA trace in Figure 1A) could be discerned. Brd2 and Brd4 showed a more discrete enrichment pattern confined to fewer regions in the LUR (Figure 1A). Several of the kLANA peaks were located in the vicinity of TSS of viral genes (Figure 1A and Table 1). Of the 12 significantly enriched kLANA peaks, 11 overlapped with Brd2 and Brd4 peaks (Figure 1A and Table 1). On comparing our kLANA ChIP-Seq data with published epigenetic modifications in BCBL-1 cells (Gunther and Grundhoff, 2010; Hu et al., 2014; Mercier et al., 2014) we noted that most regions in the KSHV genome with increased deposition of kLANA, Brd2, and Brd4 also contained the activation histone marks H3K9ac/K14ac, H3K4me3, and the facultative heterochromatin mark H3K27me3, but lacked the repressive chromatin mark H3K9me3 (Table 1). Enrichment of kLANA at the TR and at its own promoter in the LUR was consistent with the findings of previous studies (Lu et al., 2012; Kim et al., 2013; Hu et al., 2014; Mercier et al., 2014; Sun et al., 2014, 2017).

**TABLE 1:** Location of kLANA-enriched regions in the KSHV genome.

Gene	kLANA peak coordinates on KSHV genome		Distance of peak summit from TSS (bp)	Enrichment of kLANA, BRD2 and BRD4 at selected TSS						Epigenetic marks at kLANA peaks				
				kLANA		BRD2		BRD4		DNA methylation	H3K9ac/K14ac	H3K4me3	H3K9me3	H3K27me3
	Start	End		DMSO	I-BET151	DMSO	I-BET151	DMSO	I-BET151					
K6	25411	28222	−513	2.60	1.62	1.55	0.69	1.07	0.14	−	+	+	−	+
ORF32	50602	52462	−566	2.45	1.90	0.63	0.60	0.59	0.18	+	+	+	−	+
ORF40	60402	61784	−1255	2.63	1.74	0.96	0.72	0.74	0.15	+	+	+	−	+
ORF45	67647	69119	−62	2.82	1.75	0.94	0.62	0.75	0.15	+	+	+	−	+
K8.1	73069	75951	464	2.09	1.76	0.82	0.64	0.66	0.15	+	+	+	−	+
vIRF-1	82679	83768	1458	3.00	1.80	0.63	0.34	0.64	0.13	−	−	−	−	+
vIRF-1	85140	86191	−647	3.17	1.76	1.18	0.61	1.10	0.16	−	+	+	−	−
ORF58	94411	95945	−84	2.20	1.41	0.63	0.35	0.52	0.13	+	+	+	−	−
mir-K4	118948	121844	−49	2.28	2.11	0.85	0.59	0.94	0.16	−	+	+	−	+
ORF72	122138	123227	−100	2.29	1.75	0.83	0.50	0.64	0.16	+	+	+	−	+
ORF73	125536	128120	−138	2.78	2.19	0.65	0.55	0.64	0.16	−	+	+	−	−
ORF75	132565	134454	−55	2.89	1.52	1.21	0.87	1.05	0.21	+	+	+	−	−

*Peak coordinates represent the beginning and end of the kLANA peaks in the KSHV genome determined by the MACS peak caller. The numbers in these two columns refer to the nucleotide positions in the KSHV genome. Negative values for distances indicate a location of the kLANA peaks upstream of a TSS. The values for the enrichment for kLANA, BRD2, and BRD4 were calculated with respect to input. Read numbers for peak regions were extracted using the samtools ‘view’ command from both input and ChIP BAM files. The number of reads at these locations were normalized based on a linear scaling factor, which was derived from the total number of reads. Mean fold enrichment values for peak regions in ChIP samples were calculated with respect to matched input samples from the two biological replicates. In the columns ‘Epigenetic marks at kLANA peaks,’ plus and minus signs indicate an overlap or no overlap of the epigenetic mark with kLANA peaks, respectively, using data from Gunther and Grundhoff (2010), Hu et al. (2014).*

We next tested if the Brd4 inhibitor I-BET151 would interfere with the binding of kLANA to the viral genome. As expected, Brd2 and Brd4 were released from the LUR and the TR in the presence of I-BET151 (Figure 1A). While deposition of kLANA on the LUR decreased after treatment with I-BET151, kLANA remained associated with LBS1-3 in the presence of the inhibitor (Figure 1A). Conventional ChIP with primers in the TR confirmed that the deposition of Brd2 and Brd4 at loci in the TR was reduced by I-BET151 and therefore depended on their binding to acetylated histones; in contrast, binding of kLANA to the TR was not affected by the inhibitor (Figure 1B), in keeping with the direct binding of its C-terminal DNA-binding domain (DBD) to LBS1-3 DNA (Garber et al., 2002; Hellert et al., 2015).

In view of recent reports that kLANA is released from the LUR and the TR upon induction of the lytic cycle and that the Brd/BET inhibitor JQ1 is a potent inducer of the lytic cycle in PEL cells (Mercier et al., 2014; Chen et al., 2017) we investigated whether the observed release of kLANA and Brd2/4 from the LUR was accompanied by the induction of the lytic cycle by I-BET151. We treated BCBL-1 cells with increasing concentrations of I-BET151 and measured lytic reactivation by immunofluorescence assay for the early lytic marker ORF59 and by checking the expression of another lytic marker – KbZIP – on a Western blot. In comparison to the known inducers of lytic cycle SB and RTA or TPA, treatment with I-BET151 induced a very low level of lytic reactivation (Supplementary Figure S1) with less than 0.5% of cells treated with 0.5 μM I-BET151 expressing the ORF59 protein (Supplementary Figures S1A,B). We also compared the level of lytic cycle induction by I-BET151 to the previously published treatment with JQ1. The concentrations of JQ1 used by us did not induce lytic cycle activation to a high degree, although the induction was somewhat stronger than with I-BET151 (Supplementary Figures S1A–C). Since the vast majority of cells remained latent in the presence of I-BET151 it seems unlikely that lytic reactivation accounts for the decreased association of kLANA and/or Brd2/4 with the LUR following I-BET151 treatment, as observed by ChIP-Seq (Figure 1A). We therefore conclude that the interaction of kLANA with Brd2/4 (Platt et al., 1999; Viejo-Borbolla et al., 2005; Ottinger et al., 2006; You et al., 2006; Hellert et al., 2013) directly contributes to the deposition of kLANA on the 12 regions in the LUR that show an increased binding of kLANA as well as Brd2/4 (Figure 1A).

### The Association of KSHV LANA With Cellular Promoters Involves Brd/BET Proteins

**FIGURE 2:**
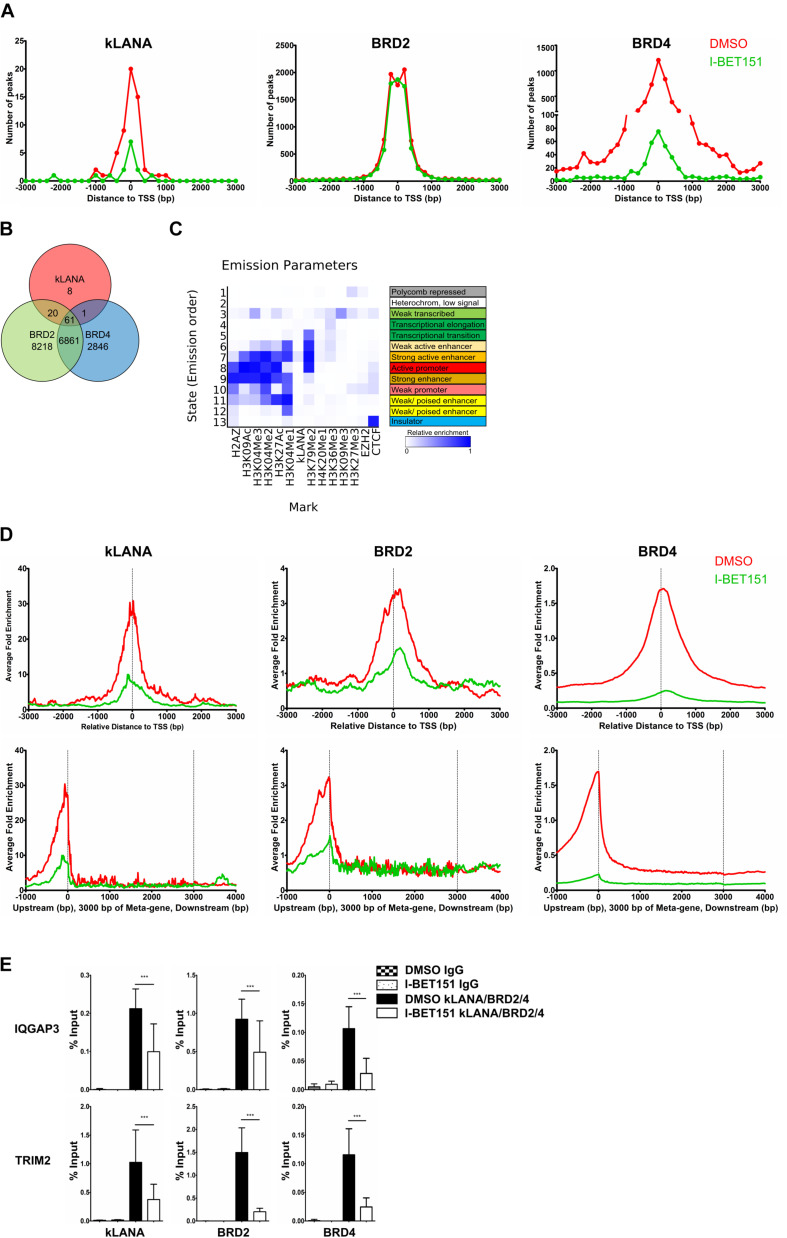
Association of kLANA with cellular TSS as a function of Brd/BET proteins. **(A)** Frequency distribution histograms of detected peaks in the absence or presence of I-BET151 relative to their distance from the TSS in bins of 200 bp. **(B)** Venn diagram showing the number of overlapping enriched regions between kLANA, Brd2, and Brd4 on the host genome. **(C)** ChromHMM analysis showing the chromatin state of kLANA-enriched TSS with epigenetic marks from the GM12878 cells derived from the ENCODE project. **(D)** Global normalized average fold enrichment profiles of ChIP-Seq reads for kLANA, Brd2, and Brd4 obtained from BCBL-1 cells that had been treated or not with I-BET151. The diagram shows the enrichment of ChIP-Seq reads in the vicinity of all cellular TSS in bins of 10 bp (top) and the global normalized average fold enrichment profiles for ChIP-Seq reads on a meta-gene of 3 kbp (bottom). **(E)** ChIP-qPCR for kLANA, Brd2, and Brd4 in I-BET151-treated/untreated BCBL-1 cells using PCR primers derived from the TSS of IQGAP3 and an intron of TRIM2. Bars represent mean ± SD. ***p < 0.001.

We also analyzed our ChIP-Seq datasets for an association of kLANA, Brd2, and Brd4 with host cell chromatin and found that all three proteins were significantly enriched at several genomic regions (Supplementary Figure S2A). We identified a total of 90 peaks for kLANA that were concordant in the two independent ChIP-Seq experiments, as well as 15160 peaks for Brd2 and 9769 peaks for Brd4. Deposition of kLANA, Brd2, and Brd4 was most prominent near cellular TSS (Figure 2A). 66% of kLANA peaks, 59% of Brd2 peaks, and 52% of the Brd4 peaks were found within ±3 kbp of the TSS (Figure 2A). This finding is consistent with the previously noted association of kLANA and Brd/BET proteins with cellular promoter regions (Lu et al., 2012; Anders et al., 2014; Hu et al., 2014; Mercier et al., 2014; Lin et al., 2017). As shown in Figure 2B, 81/90 (90%) of kLANA peaks overlapped with Brd2 peaks and 62/90 (∼ 69%) kLANA peaks overlapped with Brd4 peaks. Conversely, kLANA was only found at a small minority of Brd2 (81/15160, 0.5%) and Brd4 (62/9769, 0.6%) peaks.

We correlated our kLANA ChIP-Seq data with published histone modification data for BCBL-1 cells (Hu et al., 2014) and found that, consistent with published work (Hu et al., 2014; Mercier et al., 2014), kLANA enriched regions were often decorated with H3K4me3: 55/90 (61%) of kLANA peaks were associated with H3K4me3 (Supplementary Figure S2B). We also observed a high overlap of Brd2 and Brd4 with H3K4me3 (Supplementary Figure S2B), reflecting the enrichment of Brd2/4 in active chromatin regions (Anders et al., 2014). For a more systematic analysis of the chromatin state associated with kLANA-enriched regions, we extracted all available data for epigenetic marks from the ENCODE project for the GM12878 cell line, a B cell lymphoma cell line. We chose the datasets from the Broad Histone project due to the higher diversity of available histone modification datasets (Consortium, 2012) and used the ChromHMM tool (Ernst and Kellis, 2012) for their analysis. A total of 13 chromatin states were defined and annotated based on the observed frequency of combinational and spatial patterns of epigenetic factors (Ernst and Kellis, 2012). We observed that most kLANA-occupied regions in BCBL-1 cells were associated with the chromatin states of strong active enhancers (State 7; Figure 2C), as characterized by the combination of activation histone modifications H3K4me1, H3K4me2, H3K4me3, H3K9ac, and H3K27ac found at strong enhancers and those involved in transcriptional elongation such as H3K79me2.

Similar to the effect observed on the LUR of the viral genome, I-BET151 treatment released kLANA, Brd2, and Brd4 from the host chromatin (Figures 2A,D and Supplementary Figure S2A). This effect was apparent both when we plotted the number of detectable peaks for kLANA, Brd2, and Brd4 (Figure 2A) and their enrichment relative to the average read distribution across the human genome (Figure 2D). The bottom panel of Figure 2D illustrates the distribution of ChIP-Seq reads for kLANA, Brd2 and Brd4 across an ‘average’ gene, illustrating their preferential enrichment at transcriptional start sites (TSS). We used conventional ChIP-qPCR on selected cellular promoters of IQGAP3 and TRIM2 to confirm that the binding of kLANA as well as Brd2 and Brd4 was reduced in the presence of I-BET151 (Figure 2E). We also observed that I-BET151 displaced kLANA and Brd4 more effectively than Brd2 from both the viral and cellular chromatin (Figure 1A and Supplementary Figures S2A,D). However, we did not observe a complete release of kLANA upon I-BET151 treatment from either the KSHV or the cellular genome (Figure 1A and Supplementary Figures S2A,D), suggesting that other chromatin-associated proteins interacting with kLANA, such as DEK or MeCP2 (Krithivas et al., 2002; Griffiths and Whitehouse, 2007; Matsumura et al., 2010), or DNA motifs distantly related to LBS1-3 (Lu et al., 2012; Mercier et al., 2014), could also contribute to the association of kLANA with selected cellular TSSs.

Motif analysis of the sequences within ±75 bp from the summit of kLANA peaks showed the presence of a KSHV LBS1-like motif in 50% of the peaks (Supplementary Figure S3, top). Using CentriMo analysis, this LBS1-like motif was found to be centrally enriched within ±60 bp of kLANA peaks (Supplementary Figure S3, bottom). A FIMO scan of the entire human genome for the LBS1 motif yielded 34135 ‘hits,’ but showed that only 26.98% of them were found within ±3 kbp of a TSS. In addition, kLANA was enriched only at 0.18% of these 34135 sites (Table 2). These findings are consistent with previous work (Mercier et al., 2014). We conclude that the deposition of kLANA on the LUR of the viral genome and on the host chromatin was mediated to a significant extent by Brd2 or Brd4, but that other mechanisms, including the direct binding of kLANA to cellular DNA via its C-terminal DNA-binding domain, may also contribute.

**TABLE 2:** FIMO scan for occupancy of KSHV LBS1-like sites on the human genome by kLANA.

Cell line	Binding to TSS (%)	LBS1-like sites occupied by kLANA (%)	kLANA peaks containing LBS1-like sites (%)	kLANA peaks detectable after I-BET151 treatment (%)
BCBL-1	65.6	0.18	50	28.57
BJAB-rKSHV.219	50	0.15	59.7	17.7
BJAB-kLANA	45	2.1	7.3	43.8

*The second column shows the percent of kLANA peaks present within ±3 kbp of the TSS. Third column shows percent of KSHV LBS1-like sites occupied by kLANA on the human genome. The fourth column shows percent of total kLANA peaks on the human genome containing KSHV LBS1-like sites. Finally, the fifth column shows percent of kLANA peaks detectable after I-BET treatment.*

### Transcriptional Regulation of kLANA-Occupied Cellular Promoters

Next, we investigated the impact of kLANA binding near TSS on the transcriptional regulation of the corresponding cellular genes. In order to focus our analysis on the effects of kLANA on cellular transcription, we decided to generate a BJAB B cell line expressing kLANA alone and to compare these results to those obtained with BJAB cells. We transduced BJAB cells with a lentiviral vector expressing both kLANA and EGFP, or with the control vector expressing mCherry and EGFP, to generate, respectively, BJAB-kLANA and BJAB-mCherry. Following the enrichment of strongly EGFP expressing cells by FACS, we used bulk populations of both transduced cultures for further experiments. For comparison, we used the previously established BJAB-rKSHV.219 cell line, which had been generated by infecting BJAB cells with the GFP/RFP-expressing KSHV.219 virus (Kati et al., 2013, 2015). An immunofluorescence assay for kLANA in BJAB-kLANA cells showed diffuse nuclear staining in contrast to the KSHV-infected BJAB-rKSHV.219 cells, in which we observed the characteristic distinct kLANA speckles (Supplementary Figure S4A). Western blot analysis showed slightly stronger kLANA expression levels in the BJAB-kLANA cell line than in BJAB-rKSHV.219 cells (Supplementary Figure S4B). Both cell lines expressed the same dominant kLANA isoform; however, the molecular weight of a smaller kLANA isoform differed between BJAB-kLANA and BJAB-rKSHV.219 (Supplementary Figure S4B).

**FIGURE 3:**
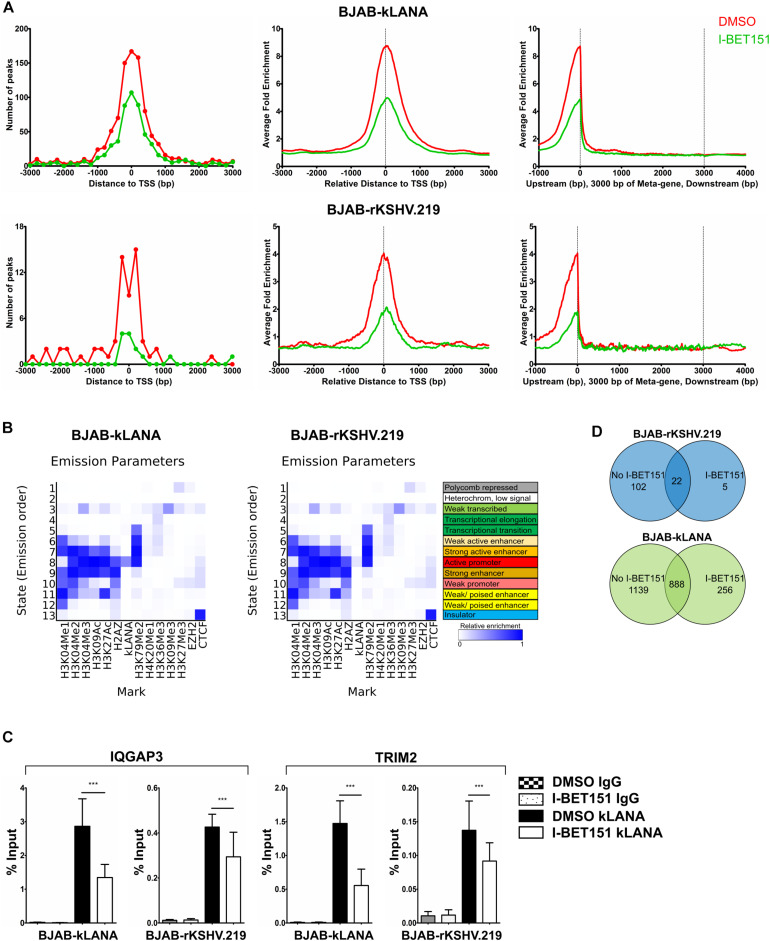
Association of kLANA with cellular chromatin in the absence or presence of the viral genome. ChIP-Seq was performed in BJAB-kLANA and BJAB-rKSHV.219 cells following treatment with 0.5 μM I-BET151 or DMSO for 48 h. **(A)** Frequency distribution histograms of kLANA ChIP-Seq detected peaks in BJAB-kLANA (top left) and BJAB-rKSHV.219 cells (bottom left) relative to their distance from the TSS in bins of 200 bp. Global normalized average fold enrichment profiles for kLANA ChIP-Seq reads in the vicinity of all cellular TSS in bins of 10 bp (top and bottom center) and on the meta-gene of 3 kbp (top and bottom right). **(B)** ChromHMM analysis identifying kLANA associated chromatin state in BJAB-kLANA cells (left) and BJAB-rKSHV.219 cells (right), using epigenetic marks from the GM12878 cells from the ENCODE project. **(C)** ChIP-qPCR for kLANA in I-BET151- or DMSO-treated BJAB-kLANA or BJAB-rKSHV.219 cells using primers derived from the TSS of IQGAP3 and an intron of TRIM2. Bars represent mean ± SD. ***p < 0.001. **(D)** Venn diagram showing overlap of kLANA ChIP-Seq read peaks in I-BET151- or DMSO-treated BJAB-rKSHV.219 (top) and BJAB- kLANA (bottom) cells.

To compare the binding of kLANA to cellular chromatin in the BJAB-kLANA and BJAB-rKSHV.219 cell lines with the pattern we had observed in PEL cells (Figure 2), we performed a ChIP-Seq experiment in the absence and presence of I-BET151. Upon aligning the ChIP-Seq reads to the host genome, we observed a higher number of kLANA peaks and stronger kLANA enrichment in BJAB-kLANA cells in comparison to BJAB-rKSHV.219 (Supplementary Figure S5A). In both cell lines, most of kLANA was enriched at or in the vicinity of TSS (Figure 3A), which was similar to the observation in BCBL-1 cells (Figures 2A,D). We identified a total of 2027 concordant peaks from two biological replicate experiments in the BJAB-kLANA cells of which 46% were found within ±3 kbp of TSS, whereas for BJAB-rKSHV.219 cells a total of 124 peaks were detected of which 50% were found within ±3 kbp of TSS (Figure 3A).

Similar to what we had observed in the BCBL-1 PEL cell line, ChromHMM analysis showed that in both the BJAB-kLANA and in BJAB-rKSHV.219 cell lines, the majority of genomic regions enriched for kLANA had epigenetic features of an active promoter enhancer state (State 8; Figure 3B), as characterized by histone modifications typical for active chromatin and transcription transition. In addition, similar to what we had found in the PEL cell line BCBL-1, motif analysis showed the presence of a centrally enriched LBS1-like site within ± 60 bp of kLANA peaks in BJAB-kLANA and BJAB-rKSHV.219 (Supplementary Figure S5B). This motif was found in 7% of the peaks in BJAB-kLANA cells and in 60% of the peaks in BJAB-rKSHV.219 cells. By performing a FIMO scan we found that similar to BCBL-1, kLANA was enriched at only 2.10 and 0.15% of regions having an LBS1-like motif in BJAB-kLANA and BJAB-rKSHV.219 cells, respectively (Table 2). Taken together, our findings indicate that kLANA shows a similar tendency to associate with a subset of transcriptionally active cellular TSS in the presence and absence of the viral genome.

Upon treatment with I-BET151, kLANA was released from the cellular chromatin of both BJAB-kLANA and BJAB-rKSHV.219 cells (Supplementary Figures S5A, 3A). Using a selected group of cellular promoters, we confirmed their reduced occupation by kLANA in the presence of I-BET151 by conventional ChIP-qPCR (Figure 3C). However, I-BET151 treatment did not result in a complete release of kLANA in either BJAB-kLANA or BJAB-rKSHV.219 (Supplementary Figures S5A, 3C). In BJAB-rKSHV.219 cells we found 124 kLANA peaks, of which 22 (18%) were still detectable after I-BET151 treatment (Figure 3D, top), indicating that, comparable to the result obtained in the PEL cell line (Figure 2B), the majority of kLANA peaks in this KSHV-infected cell line depended on its interaction with Brd/BET proteins. In contrast, we could still detect 888 of 2027 kLANA peaks (44%) in BJAB-kLANA cells after I-BET151 treatment, reflecting the broader distribution of kLANA in this cell line (Figure 3D, bottom). We also observed 5 regions in BJAB-rKSHV.219 and 256 regions in BJAB-kLANA cells that had an increased enrichment in the presence of I-BET151 (Figure 3D). These results indicate that, as in PEL cells (Figure 2B), kLANA is preferentially associated with promoters showing active transcription marks in *in vitro* infected BJAB-rKSHV.219 cells as well as in kLANA-transduced BJAB, and Brd/BET proteins contribute to the kLANA clustering near the TSS.

**FIGURE 4:**
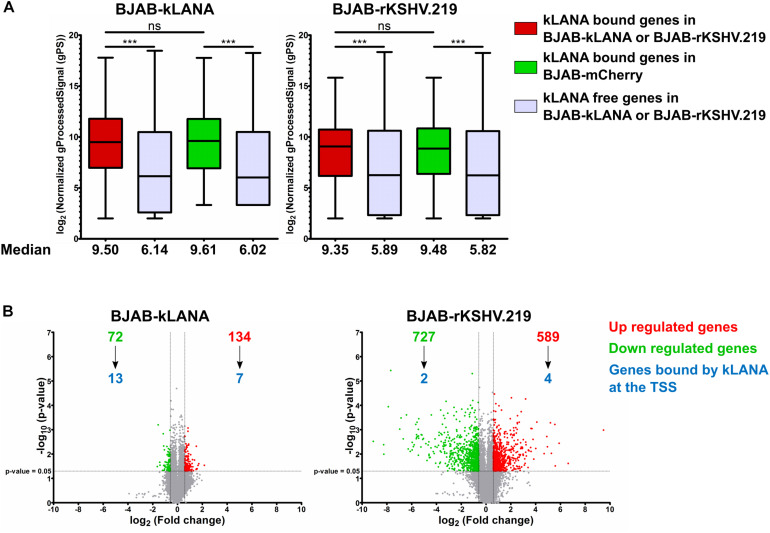
kLANA-mediated cellular transcriptional regulation. Total RNA was isolated from BJAB-mCherry, BJAB-kLANA and BJAB-rKSHV.219 cells and used for transcriptome analysis. The plotted expression levels are relative and normalized log_2_ transformed values. For identifying differentially regulated genes the filtering criteria used were 1.5 fold change and a *p*-value less than 0.05. **(A)** Box plot for comparison of expression levels between kLANA bound and kLANA-free genes in BJAB-kLANA (left) or BJAB-rKSHV.219 (right) and BJAB-mCherry cells. Red boxes indicate kLANA-bound genes in kLANA positive cells (BJAB-kLANA or BJAB-rKSHV.219) and green boxes indicate the same genes in kLANA negative BJAB-mCherry cells. Gray boxes indicate genes not bound by kLANA. Boxes represent interquartile range with Min-Max whiskers. ***p < 0.001. **(B)** Volcano plots for differential regulation of all cellular genes in BJAB-kLANA (left) and BJAB-rKSHV.219 (right) cells. Green numbers or dots represent downregulated genes, red numbers or dots represent upregulated genes, and gray dots indicate genes that are not differentially regulated. The numbers of genes bound by kLANA at their TSS are marked in blue.

We then asked if the association of kLANA with active promoter or strong active enhancer chromatin states could have an impact on the transcriptional regulation of the corresponding cellular genes. To this end, we compared the transcriptome of BJAB-kLANA, BJAB-rKSHV.219, and BJAB-mCherry cells as control, using a genome-wide gene expression array. We extracted the normalized intensity values obtained with the gene expression array for genes bound by kLANA at or in the vicinity ±3 kbp of their TSS. For both BJAB-kLANA and BJAB-rKSHV.219, we observed that cellular genes, whose TSS were occupied by kLANA were transcribed at a higher median level than those not bound by kLANA (Figure 4A). To understand whether the higher level of transcription for kLANA occupied genes was a result of kLANA activating their transcription or due to the fact that kLANA preferentially associated with the TSS of genes that were already transcriptionally active, we compared the expression levels of cellular genes, that were occupied by kLANA in BJAB-kLANA or BJAB-rKSHV.219 with the expression levels of the same genes in BJAB-mCherry. We observed that genes whose promoters were occupied by kLANA in BJAB-rKSHV.219 or in BJAB-kLANA cells showed, on average, similar transcription levels in KSHV-infected or kLANA-transduced BJAB as in the control BJAB-mCherry cells, supporting the interpretation that kLANA associates with already transcriptionally active promoters (Figure 4A). To identify cellular genes that are occupied and transcriptionally regulated by kLANA, we calculated the fold change of gene expression in BJAB-kLANA and BJAB-rKSHV.219 in comparison to BJAB-mCherry cells. Comparing BJAB-kLANA with BJAB-mCherry cells, we observed a total of 206 differentially regulated genes, of which 134 were upregulated and 72 were down regulated (Figure 4B). Only 7 of the 134 upregulated and 13 of the 72 downregulated genes have kLANA deposited near their TSS (Figure 4B). Comparing BJAB-rKSHV.219 cells with BJAB-mCherry cells, we observed a total of 1316 differentially regulated genes, of which 589 were upregulated while 727 were downregulated. Again, only a minority of the differentially expressed cellular genes (4/589 upregulated and 2/727 downregulated) had kLANA deposited near their TSS (Figure 4B). The list of up- and downregulated genes bound by kLANA at the TSS in the respective cell lines is presented in Supplementary Table S1. This analysis suggests that more cellular genes are differentially regulated in KSHV-infected vs. kLANA only expressing cells probably as a result of viral factors other than kLANA impacting cellular transcription. Our results also suggest that only a small percentage of differentially expressed genes are likely to be up- or downregulated as a result of kLANA binding to their TSS. With respect to a role of either Brd2- or Brd4-dependent binding of kLANA on TSS in the transcriptional regulation of the corresponding genes, we found 20 regions at the TSS of host genes that overlap in our ChIP-Seq datasets for kLANA (in BCBL-1, BJAB-kLANA, and BJAB-rKSHV.219) and Brd2 (in BCBL-1), and 18 regions that overlap between kLANA and Brd4 (in BCBL-1) (Supplementary Table S1). On correlating the ChIP-Seq with the transcriptomics data we identified only 5 genes whose TSS are occupied by kLANA-Brd2 or kLANA-Brd4 and whose expression is regulated by kLANA. Interestingly, the TSS of all five genes (DNMT3A, FBXO4, HECTD2, NIPAL2, and NME6) are occupied by both Brd2 and Brd4. In BJAB-kLANA cells HECTD2 and NIPAL2 are downregulated. In BJAB-rKSHV.219 cells, DNMT3A, and NME6 are upregulated and FBXO4 is downregulated. Therefore, only 2 genes in BJAB-kLANA cells and only 3 genes in BJAB-rKSHV.219 cells could be considered to be differentially regulated due to kLANA-Brd2/4 binding at their TSS. These genes are marked in bold in the Supplementary Table S1. As suggested previously (Mercier et al., 2014), kLANA may therefore influence key regulatory genes that are at the hub of or lie upstream in the regulatory networks.

In a separate attempt to explore the role of Brd2/4 in kLANA-mediated cellular transcriptome changes, we treated BJAB-kLANA and BJAB-rKSHV.219 cells with 0.5 μM I-BET151 and measured the transcriptome changes by gene expression array. We observed that some cellular genes, whose TSS are occupied by kLANA, show a reduced expression following I-BET151 treatment. For example, in I-BET151-treated BJAB-kLANA cells, we found IQGAP3 and TRIM2 – occupation of their TSS by LANA and Brd2/4 can be inhibited by I-BET151 (Figure 2E) – to be downregulated by a factor of 2.63 and 2.83, respectively. Likewise, in I-BET151-treated BJAB-rKSHV.219 cells, IQGAP3, and TRIM2 gene expression was downregulated by I-BET151 with a factor of 2.10 and 3.11, respectively. However, as previously reported by others (Dawson et al., 2011; Chaidos et al., 2014; Yoshida et al., 2015; Tyler et al., 2017; Trivedi et al., 2020), BET inhibitors induce very extensive transcriptome changes and we therefore feel that it may be difficult to link cellular genes, whose expression is regulated by I-BET151, to those whose TSS are occupied by kLANA.

### The Association of MHV-68 mLANA With Cellular Promoters Involves Brd/BET Proteins

Murine gammaherpesvirus MHV-68 is phylogenetically related to KSHV and EBV and has been used extensively to study the replication of, and immune response to, a gammaherpesvirus in a murine infection model. Similar to KSHV, its ORF73 codes for an origin binding protein (mLANA), which is important for latency establishment and viral episome persistence (Fowler et al., 2003; Habison et al., 2012). Its N-terminal end contains a chromatin binding domain and its C-terminal DNA-binding domain (DBD) shows sequence and structural homology to the KSHV and EBV DBDs and binds directly to the MHV-68 TR (Habison et al., 2012; Correia et al., 2013; Hellert et al., 2013). mLANA also binds to Brd/BET proteins (Ottinger et al., 2009). An mLANA mutant deficient in binding to the Brd/BET proteins is unable to mediate latent persistence *in vivo* (Hellert et al., 2013). We therefore investigated if the mLANA-chromatin association is also mediated by Brd/BET proteins.

**FIGURE 5:**
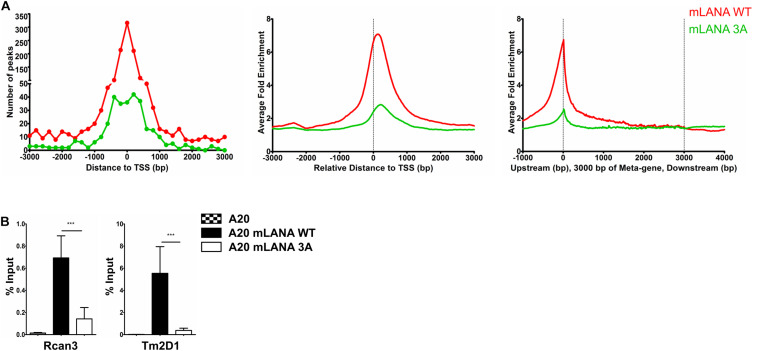
Brd/BET-mediated chromatin association of mLANA. ChIP-Seq was performed in A20-GFP, A20-mLANA WT, and A20-mLANA 3A cells that were treated with doxycycline at a final concentration of 1 μg/ml for 48 h. **(A)** Frequency distribution histograms of mLANA WT or mLANA 3A enriched regions relative to their distance from the TSS in bins of 200 bp (left), global normalized average fold enrichment profiles for mLANA WT or mLANA 3A in the vicinity of all cellular TSS in bins of 10 bp (center) and global normalized average fold enrichment profiles for mLANA WT or mLANA 3A on the meta-gene of 3 kbp (right). **(B)** ChIP-qPCR for mLANA WT and mLANA 3A mutant at the TSS of Rcan3 and Tm2D1. Bars represent mean ± SD. ***p < 0.001.

Since *de novo* infection of cultured cells with MHV-68 does not result in the establishment of latency, no comparison of a latently MHV-68 infected cell with its uninfected counterpart is possible. We therefore transduced mouse A20 B cells with lentiviral vectors to express a FLAG-tagged mLANA WT or the previously described mLANA 3A mutant (K169A, K224A, and K228A), which is deficient in binding to Brd2 and Brd4 *in vitro* (Hellert et al., 2013), in a doxycycline-inducible manner (Supplementary Figure S6A). We used these cell lines, after treatment with doxycycline for 48 h, for a mLANA ChIP-Seq experiment using an antibody to the FLAG epitope. After aligning the reads to the mouse genome, we observed several genomic regions that were enriched for mLANA WT and/or the mLANA 3A mutant (Supplementary Figure S6B). We detected a total of 6138 peaks for mLANA WT, of which 1352 were detected within ±3 kbp of the TSS. For mLANA 3A, 698 peaks were identified, of which 319 peaks were detected within ±3 kbp of the TSS (Figure 5A). We chose two mLANA-occupied cellular promoters (for Rcan3 and Tm2D1) and compared their association with mLANA WT and the mLANA 3A mutant by conventional ChIP-PCR. We found that they are indeed associated with mLANA WT, but much less with the Brd2/4-binding deficient mLANA 3A mutant (Figure 5B). These findings suggest that the recruitment of mLANA to TSS is at least partially due to its interaction with cellular Brd/BET proteins.

### Impact of the Brd/BET-Protein-Dependent Association of mLANA With TSS on Cellular Gene Expression

**FIGURE 6:**
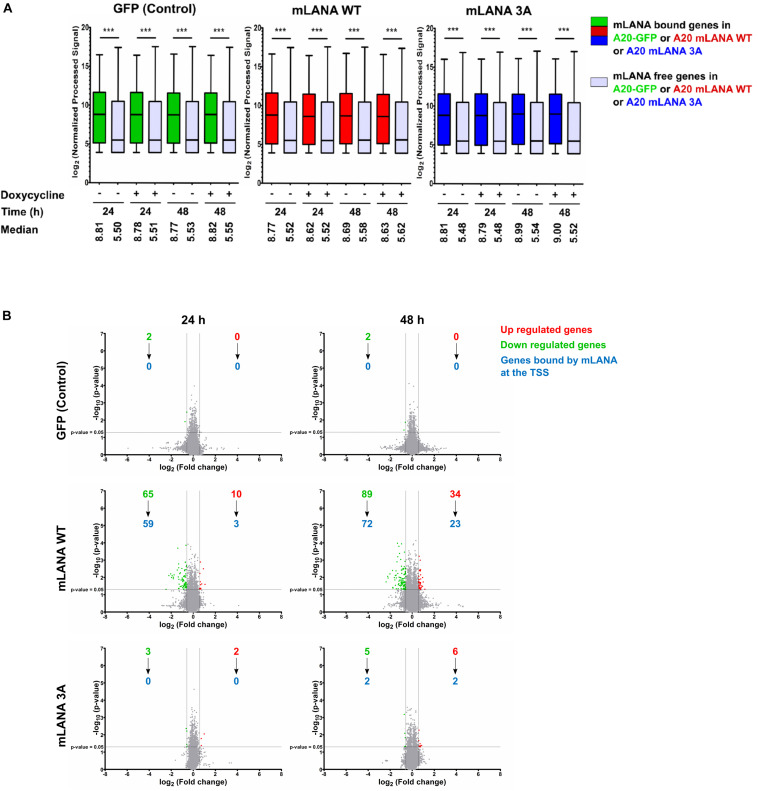
mLANA-mediated cellular transcriptional regulation. **(A)** Box plots of gene expression microarray signals for cellular genes in mLANA WT-, mLANA 3A-, or GFP-expressing A20 cells. The panel shows a comparison of expression levels for cellular genes, whose TSS are occupied by mLANA or not. Red boxes indicate cellular genes, whose TSS are occupied by mLANA in mLANA WT expressing cells, green boxes indicate the same genes in the A20-GFP control cells and blue boxes the same genes in mLANA-3A expressing cells. Gray boxes indicate genes not bound by mLANA. Boxes represent interquartile range with Min-Max whiskers. ***p < 0.001. **(B)** Volcano plots for differential regulation of all cellular genes by mLANA WT vs. mLANA 3A for two time points after induction of mLANA expression by doxycycline. Values or dots in green or red represent downregulated and upregulated genes, respectively, gray dots genes that are not differentially regulated. Blue numbers indicate cellular genes, whose TSS are occupied by mLANA in each category.

Since mLANA, similarly, to kLANA, also showed a Brd/BET dependent association with cellular TSS, we explored its impact on cellular gene expression. We performed a gene expression microarray experiment with A20 murine B cell lines expressing mLANA WT, the mLANA 3A mutant or GFP only, after treatment with doxycycline for 24 and 48 h. We then correlated the gene expression data with the mLANA ChIP-Seq data. We compared the expression levels of genes bound by mLANA WT at or in the vicinity of their TSS with those not bound by mLANA WT. Similarly, to kLANA, mLANA WT-occupied genes showed a higher median level of expression in comparison to unoccupied genes. This phenomenon was independent of doxycycline treatment and expression levels of these genes in the control A20-GFP cells showed that these genes were already transcriptionally active in the absence of mLANA. These findings indicate that mLANA WT associates with transcriptionally active chromatin (Figure 6A). We then calculated, for the A20-GFP, mLANA WT, and mLANA 3A cell lines and each gene, the ratio of the array signals for the doxycycline-induced to uninduced samples to identify genes that are differentially regulated by mLANA. For the A20-GFP control cells, treatment with doxycycline downregulated only two cellular genes, indicating that the effect of doxycycline on transcription was negligible in this experiment. In the case of mLANA WT, a total of 75 genes were differentially regulated after 24 h of doxycycline treatment, of which 10 were up- and 65 were downregulated. This number increased to 123 genes, of which 89 were down- and 34 were upregulated after 48 h of doxycycline treatment (Figure 6B). Correlating these results with ChIP-Seq data showed that ∼90% (59/65) of the genes downregulated at 24 h, and ∼80% (72/89) of the genes downregulated at 48h, were bound by mLANA WT. At 48 h, we also found approximately 67% (23/34) of upregulated genes to have mLANA-occupied TSS (Figure 6B). In contrast, there were significantly fewer differentially regulated genes in the cell line expressing the mLANA 3A mutant, with only 5 genes differentially regulated at the 24 h time point (3 down, 2 up) and 11 (5 down, 6 up) at the 48 h time point. Among the cellular genes whose expression we found to be downregulated by mLANA WT but not by mLANA 3A and for which we found deposition of mLANA WT, but not mLANA 3A, near their TSS, were Rcan3 and Tm2D1 (Figure 5B) as well as C2cd2, Endod1, Fam92a, Fbxo4, FoxA1, Hectd2, Ogfrl1, Trdmt1, and Ubtd2. This indicated that Brd/BET protein-dependent association of mLANA with TSSs may result in either transcriptional repression or activation. However, since our ChIP-Seq data showed a total of 6138 mLANA-enriched regions, of which 1351 were within ±3 kbp of TSS, whereas we observed only 123 differentially regulated genes after 48 h of doxycline induction, it appears that mLANA-dependent transcriptional regulation occurs only on a subset of mLANA occupied cellular genes.

## Discussion

Members of the cellular Brd/BET protein family are epigenetic readers that recognize specific histone acetylation patterns that are typical for transcriptionally active chromatin by virtue of the two bromodomains (Florence and Faller, 2001; Zeng and Zhou, 2002) and recruit cellular factors with the help of their ET domains to modulate gene expression (Dhalluin et al., 1999; Platt et al., 1999; Denis, 2001; Yang et al., 2005; Denis et al., 2006; Peng et al., 2007; Rahman et al., 2011; LeRoy et al., 2012; Anders et al., 2014; Fujisawa and Filippakopoulos, 2017). Brd/BET proteins have also been shown to modulate the productive replication of several DNA viruses, including human papillomavirus HPV16 and the Merkel cell polyomavirus, and to regulate transcription of the HIV LTR, the human cytomegalovirus and KSHV LANA (Stedman et al., 2004; Kapasi and Spector, 2008; Wang et al., 2012, 2013; Boehm et al., 2013; Hellert et al., 2013; Li et al., 2013; Chen et al., 2017). Furthermore, Brd2 and Brd4 have been shown to direct the choice of integration sites near cellular TSS in the case of the γ-retroviruses MLV and FeLV (Gupta et al., 2013; Sharma et al., 2013; Gogol-Doring et al., 2016) and Brd4 serves as an attachment site for BPV episomes on host mitotic chromatin (You et al., 2004, 2005; McPhillips et al., 2005). The current overall picture of the role of Brd/BET proteins in the regulation of viral replication, gene expression and genome integration is therefore one of cellular factors that can interact with several viral proteins such as KSHV kLANA and MHV68 mLANA, EBV EBNA1, BPV and HPV E2, MCV LT antigen, as well as MLV and FeLV integrase; Brd2/4 can direct these viral proteins and, in the case of viral origin-binding proteins HPV and BPV E2 or the MLV or FeLV integrases, the viral genome or pre-integration complex to particular sites in the cellular genome that are favorable for viral replication, viral genome maintenance or integration. The best characterized examples include Brd4-mediated attachment of the BPV E2 protein and viral episome to mitotic chromatin, the involvement of Brd4 in directing the HPV16 genome to fragile sites in the cellular genome, which facilitates viral vegetative DNA replication, and the role of Brd2/4 in directing the MLV pre-integration complex to cellular TSS and thereby determining the typical MLV integration pattern near cellular TSS.

Although kLANA was the first viral protein to be reported as an interactor of a Brd/BET family member (Platt et al., 1999), the role of these epigenetic modulators in the life cycle of gammaherpesviruses remains unclear. Previous report suggested that Brd4 may contribute to the association of kLANA with mitotic chromatin (You et al., 2006) and that Brd2 or Brd4 may be involved in kLANA-mediated transcriptional regulation of a gene involved in cell cycle regulation (Viejo-Borbolla et al., 2005; Ottinger et al., 2006). The interaction of EBV EBNA1 with Brd4 has been linked to transcriptional control (A. Lin et al., 2008). A recombinant MHV68 carrying an mLANA mutant deficient in Brd2/4 binding has been reported to be incapable of establishing latency in the spleen of infected mice, but other Brd2/4-binding deficient mLANA mutants did not show this phenotype (Correia et al., 2013; Hellert et al., 2013).

In this study we therefore explored (i) if the interaction of kLANA with Brd2 or Brd4 contributes to the previously reported preferential deposition of kLANA on cellular TSS and, (ii) if so, whether this would have an impact on kLANA-mediated transcriptional regulation. We also analyzed whether mLANA shows any similarities to kLANA with regards to genomic distribution and contribution to transcriptional regulation. Using a ChIP-Seq approach we confirmed that, on the viral genome, LANA is deposited on the LUR in addition to binding to the TR subunits in a LBS-sequence-dependent manner. The distribution of kLANA on the KSHV LUR is broad, but peaks in several regions that are also occupied by Brd2 and Brd4 (Figure 1A and Table 1). Interestingly, the number of kLANA ChIP-Seq reads mapping to these peaks in the KSHV LUR is reduced in I-BET151-treated cells along with, but not as dramatically as is the case for the number of Brd4 ChIP-Seq reads (Figure 1A). This observation suggests that the binding of kLANA to Brd4 may contribute to its association with the KSHV LUR. In contrast to Brd4, Brd2 deposition on the KSHV LUR is less markedly reduced in I-BET151-treated cells, in keeping with the observation that I-BET151 is mainly targeting the first bromodomain of Brd4 (Dawson et al., 2011; Tyler et al., 2017). The residual association of kLANA with the KSHV LUR in I-BET151-treated cells could therefore be mediated by Brd2, or alternatively by other cellular chromatin proteins such as MeCP2 or DEK, with which kLANA has also been shown to interact (Krithivas et al., 2002; Matsumura et al., 2010).

We further found that kLANA is preferentially associated with cellular TSS, as reported previously (Lu et al., 2012; Hu et al., 2014; Mercier et al., 2014; Lin et al., 2017). Interestingly, treatment with I-BET151 markedly reduced the deposition of kLANA, as well as of Brd4 and Brd2, on cellular TSS, suggesting that the previously reported interaction of kLANA with Brd2 and Brd4 (Platt et al., 1999; Viejo-Borbolla et al., 2005; Ottinger et al., 2006; You et al., 2006) contributes to this characteristic association of kLANA with cellular TSS. We observed this reduction of kLANA deposition on cellular TSS in a naturally latent PEL cell line (Figures 2A,D,E and Supplementary Figure S2A), as well as in a BJAB cell line that had been infected with KSHV *in vitro* and requires continuous selection with puromycin to maintain the KSHV genome (BJAB-rKSHV.219; Figure 3A). Similarly, a kLANA-expressing BJAB cell line (BJAB-kLANA) in which LANA localizes diffusely in the nucleus showed the same deposition of kLANA on cellular TSS which could be reduced by treatment with I-BET151 (Figure 3A). This suggests that the interaction of kLANA with Brd2/4 is probably not sufficient to explain the stable maintenance of KSHV genomes in a PEL cell line and that the Brd2/4-dependent deposition of kLANA on cellular TSS may be due to ‘free’ (i.e., not associated with LANA speckles) nuclear kLANA. Although superficially the deposition of kLANA near cellular TSS resembles the Brd2/4-dependent typical integration pattern of the γ-retrovirus MLV (De Rijck et al., 2013; Gupta et al., 2013; Gogol-Doring et al., 2016), it therefore need not imply a preferential localization of KSHV episomes near TSS. When comparing ChIP-Seq results between the different cell lines, we noticed that the host genomes of KSHV positive cells, BCBL-1 and BJAB-rKSHV.219, had lower number of total kLANA peaks than the BJAB-kLANA cells (Figures 2B,D). This possibly suggests that the viral genome absorbs kLANA molecules, so that fewer are available to bind to the cellular genome.

To extend these observations to the related γ2-herpesvirus MHV-68 and to control for the well-known pleiotropic effects of I-BET151 on cellular gene expression (Nicodeme et al., 2010; Long et al., 2014; Wyspianska et al., 2014; Tyler et al., 2017), we generated stable A20 murine B cell lines expressing either mLANA WT or mLANA mutant, in which three lysine residues in the basic patch on the surface of the mLANA DNA-binding domain had been mutated to alanine residues and which had lost the ability to interact with Brd2 and Brd4 (Hellert et al., 2013). Similarly, to kLANA, mLANA is preferentially associated with cellular TSS and this association is markedly reduced in the case of the mLANA 3A mutant (Figure 5 and Supplementary Figure S6B). Taken together, the results obtained with KSHV-infected or kLANA-expressing cells treated with I-BET151, and mLANA WT- or mLANA 3A-expressing cells point to a role for members of the Brd/BET family of epigenetic regulators in tethering kLANA and mLANA to cellular TSS. However, the observation that I-BET151 treatment or the use of the mLANA 3A mutant did not completely abolish the association of kLANA or mLANA with cellular TSS (Figures 2A,D,E, 3A,C, 5) and the fact that we could identify LBS1-like DNA sequence consensus motifs in cellular TSS, as reported previously (Lu et al., 2012; Mercier et al., 2014; Lin et al., 2017), suggest that binding to Brd2/4 does not explain all the association of kLANA or mLANA with cellular TSS. Likewise, only a small percentage of kLANA-occupied TSS showed the LBS-like consensus motif depicted in Supplementary Figures S3, S5B and Table 2. Additionally, the results of the FIMO scan in the BJAB-kLANA cell line stood out with regard to lower number of kLANA binding sites containing LBS1-like motifs (Table 2). These data suggest that in the absence of viral DNA kLANA binding to cellular genome might take place to a higher extent through chromatin associated proteins, such as Brd/BET proteins, while in the presence of the viral genome, significant amount of kLANA associates with cellular chromatin through the LBS1-like motifs. It is tempting to hypothesize that there could be two ‘states’ of kLANA, one allowing direct binding to DNA and another optimal for binding to chromatin through other proteins.

We next addressed the question if the binding of kLANA or mLANA to cellular TSS could have an impact on their transcriptional regulation of the corresponding cellular genes. Cellular TSS occupied by kLANA showed an enrichment of epigenetic markers characteristic of active enhancers or promoters (Figures 2C, 3B and Supplementary Figure S2B) and belonged to cellular genes with high basal mRNA expression levels (Figures 4A, 6A). However, most cellular TSS occupied by kLANA in KSHV-infected or kLANA-expressing B cells did not belong to cellular genes whose transcription was up- or downregulated in the presence of the KSHV genome or kLANA (Figure 4B). For mLANA-expressing murine B cells we found more cellular genes to be up- or downregulated compared to cells expressing the mLANA 3A mutant that is deficient in the interaction with Brd2 and Brd4 and which showed a reduced deposition of mLANA on cellular TSS (Figures 5, 6B); in addition, a significant proportion of mLANA-upregulated or -downregulated cellular genes had TSS that were occupied by mLANA (Figure 6B). This observation suggests that the ability of mLANA to modulate the transcription of cellular genes in A20 B cells may at least in the case of some genes depend on its Brd2/4-dependent association with cellular TSS. However, we cannot exclude the possibility that altering the overall charge of the basic patch region in the mutant mLANA DBD by introducing three lysine to alanine changes might have an independent impact on cellular gene expression.

We also explored a possible link between kLANA binding to cellular TSS and cellular transcriptome changes in I-BET151-treated cells, but found the analysis of massive transcriptional changes induced by I-BET151 to render kLANA-dependent effects difficult to disentangle. Overall, our findings suggest that, similar to their role in determining the typical γ-retroviral integration pattern near cellular TSS and locating HPV genomes to cellular genomic regions associated with fragile sites, Brd/BET proteins appear to locate kLANA and mLANA near cellular TSS. At least in KSHV-infected cells the deposition of kLANA on a selected group of TSS may not be sufficient on its own to result in a transcriptional regulation of the corresponding cellular genes, but may facilitate the role of additional transcriptional regulators or pathways. In a mLANA-expressing B cell line we found evidence in support of the interpretation that, in some cases, the recruitment of mLANA to cellular TSS by Brd2, Brd4 or another member of the Brd/BET family might impact cellular transcription.

## Data Availability Statement

The datasets generated in this study can be found in online repositories. The names of the repository/repositories and accession number(s) can be found below: https://www.ncbi.nlm.nih.gov/geo/query/acc.cgi?acc=GSE153244.

## Author Contributions

TFS and MW-G: conceptualization. TFS: funding acquisition. RL, US, and MW-G: investigation. RL, US, and MP: resources. RL and TG: methodology. RL, TG, AG, and MW-G: analysis. RL and TG: validation. RL: project administration and writing – original draft. RL and MW-G: visualization. TFS, MW-G, and AG: supervision. TFS, RL, and MW-G: review and editing. All authors contributed to the article and approved the submitted version.

## Conflict of Interest

The authors declare that the research was conducted in the absence of any commercial or financial relationships that could be construed as a potential conflict of interest.

## Footnotes

1 https://www.ncbi.nlm.nih.gov/geo/query/acc.cgi?acc=GSE153244
