# Supplementary figures and images for: Brd/BET Proteins Influence the Genome-Wide Localization of the Kaposi’s Sarcoma-Associated Herpesvirus and Murine Gammaherpesvirus Major Latency Proteins
Source: Front Microbiol. 2020 Oct 22;11:591778. doi: 10.3389/fmicb.2020.591778 (PMC7642799; doi:10.3389/fmicb.2020.591778)

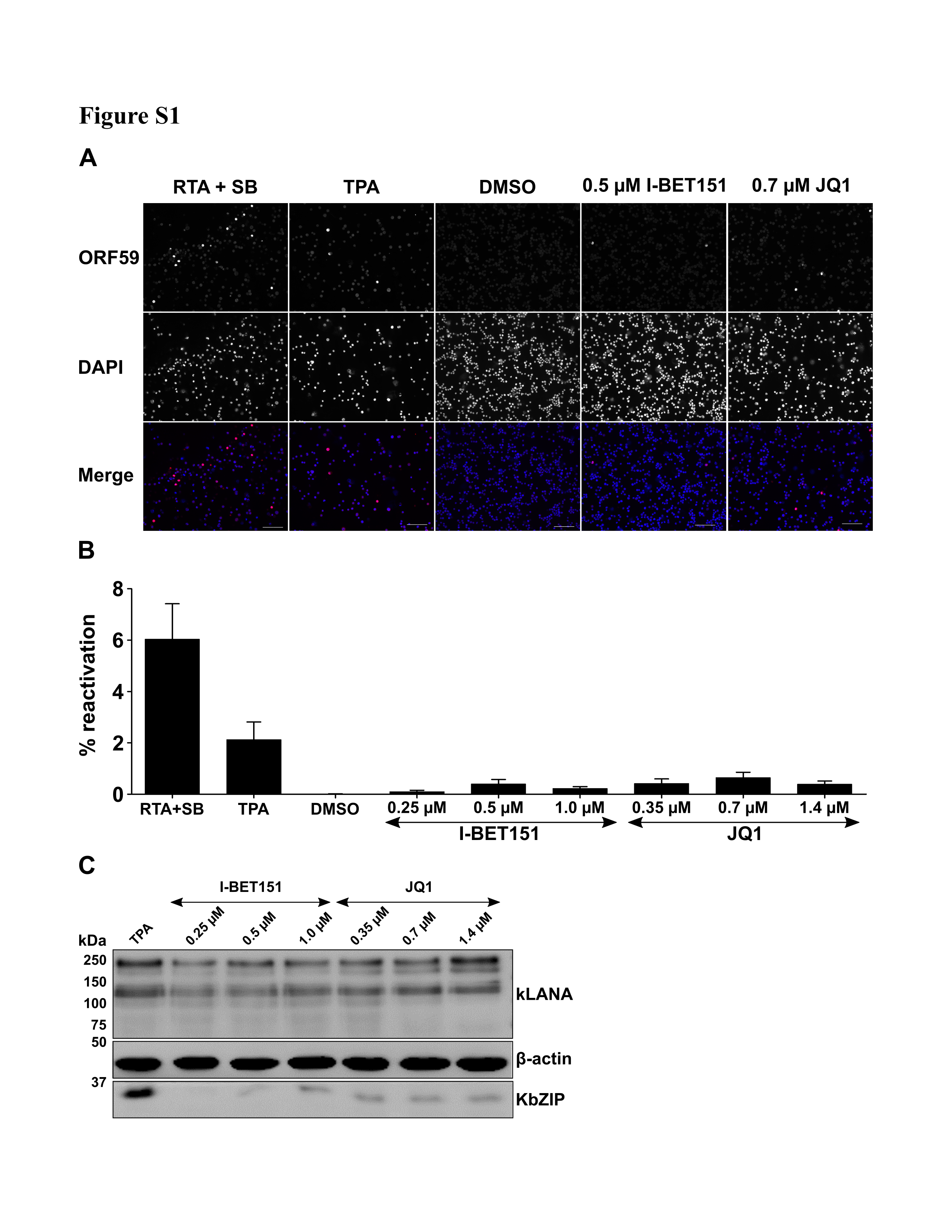

Supplement: Supplementary file 1 [file Image_1.TIFF]

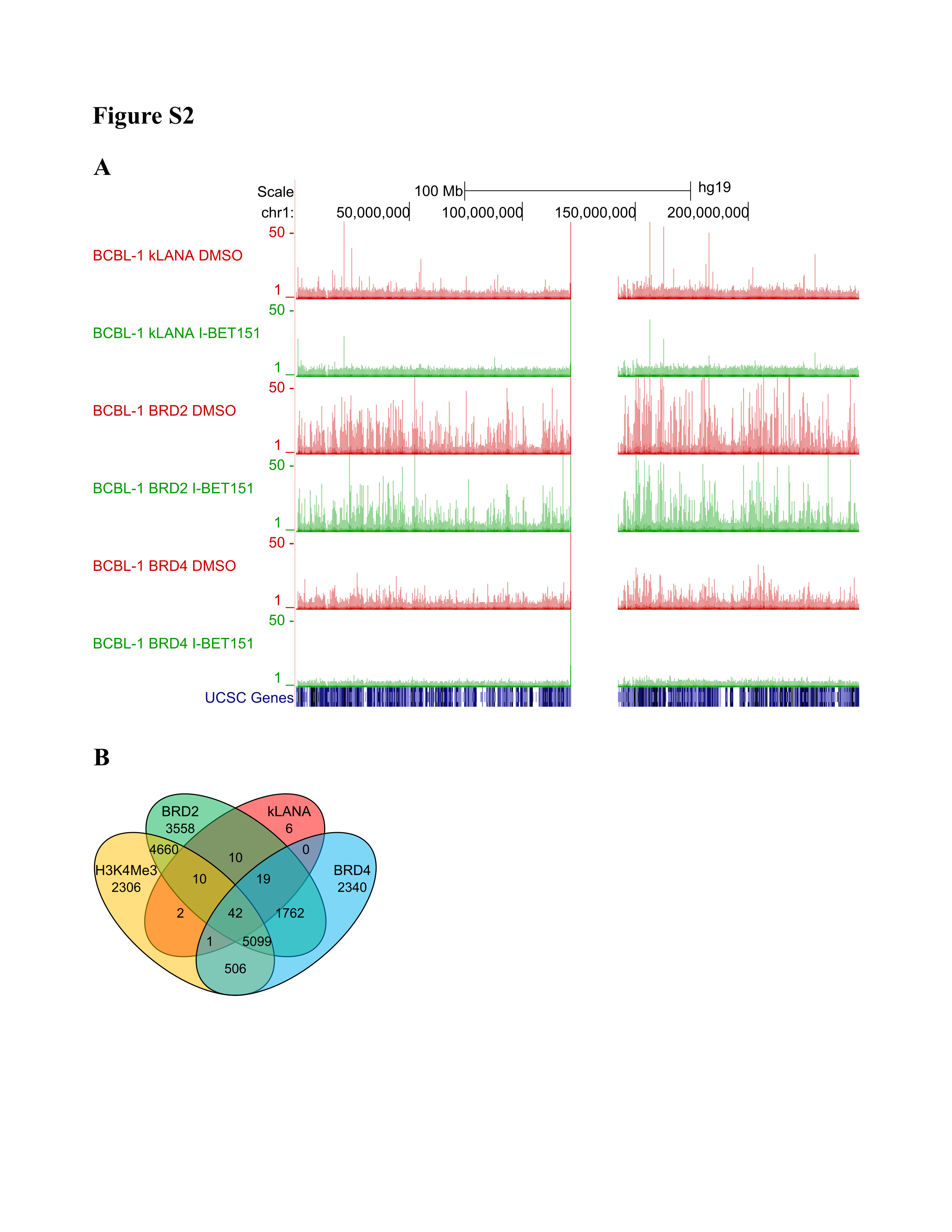

Supplement: Supplementary file 2 [file Image_2.TIFF]

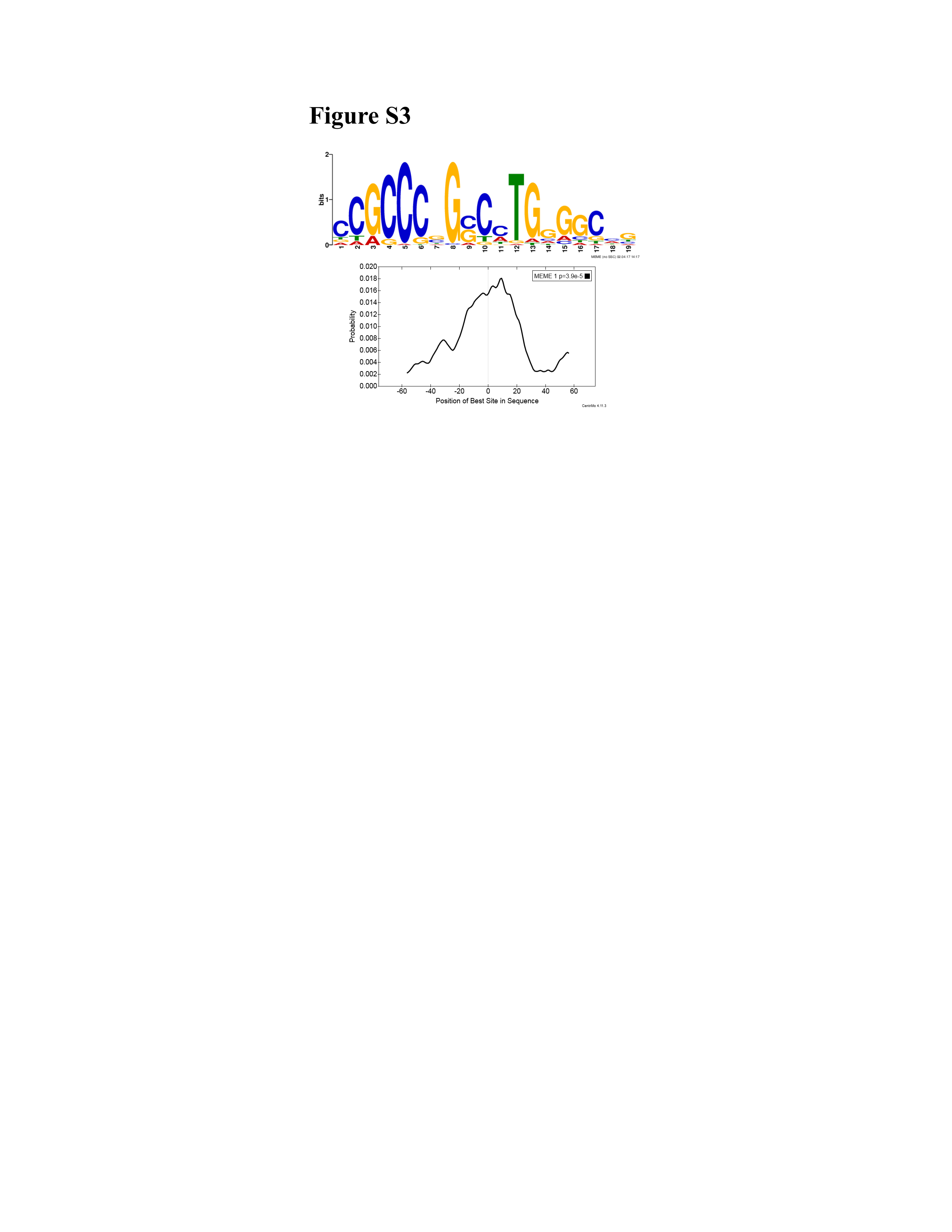

Supplement: Supplementary file 3 [file Image_3.TIFF]

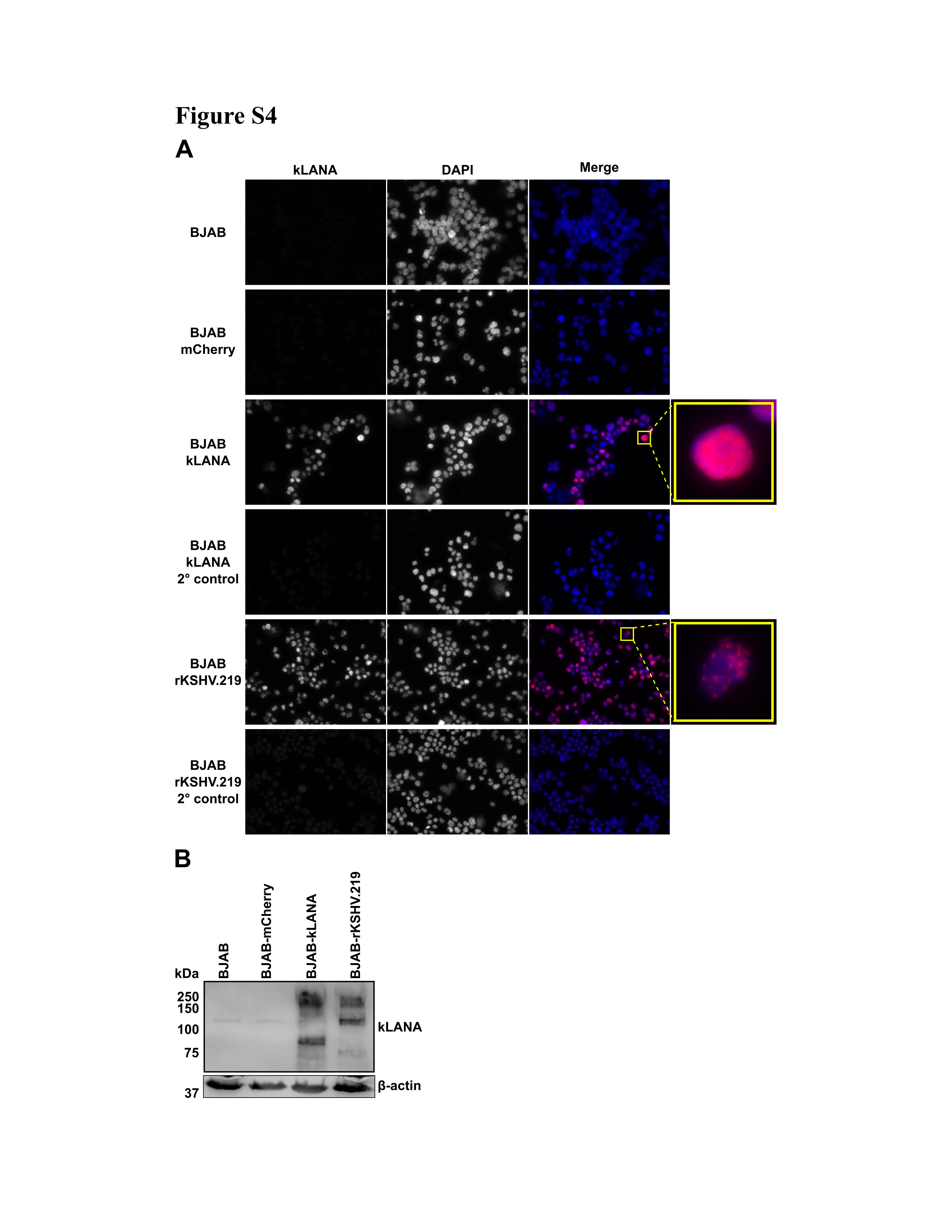

Supplement: Supplementary file 4 [file Image_4.TIFF]

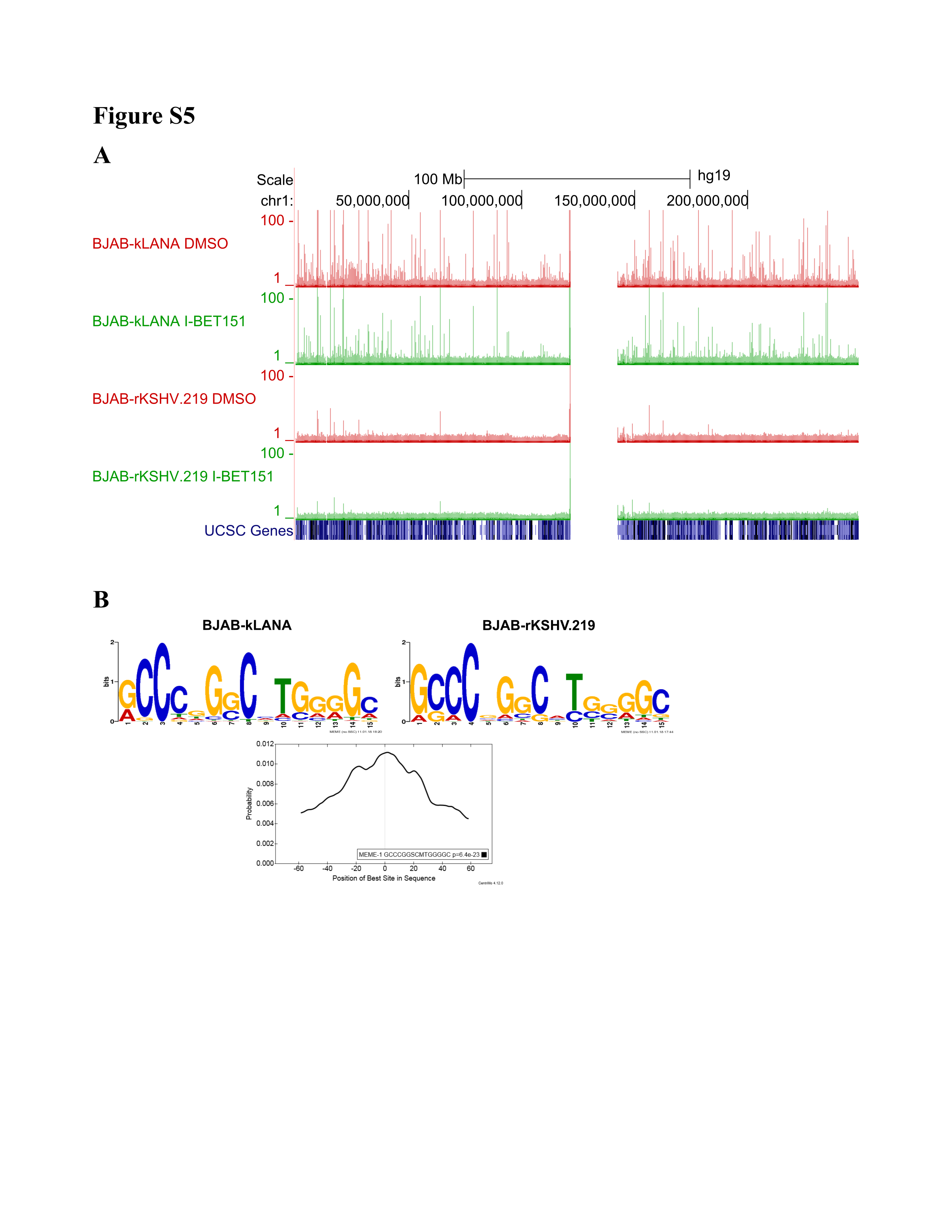

Supplement: Supplementary file 5 [file Image_5.TIFF]

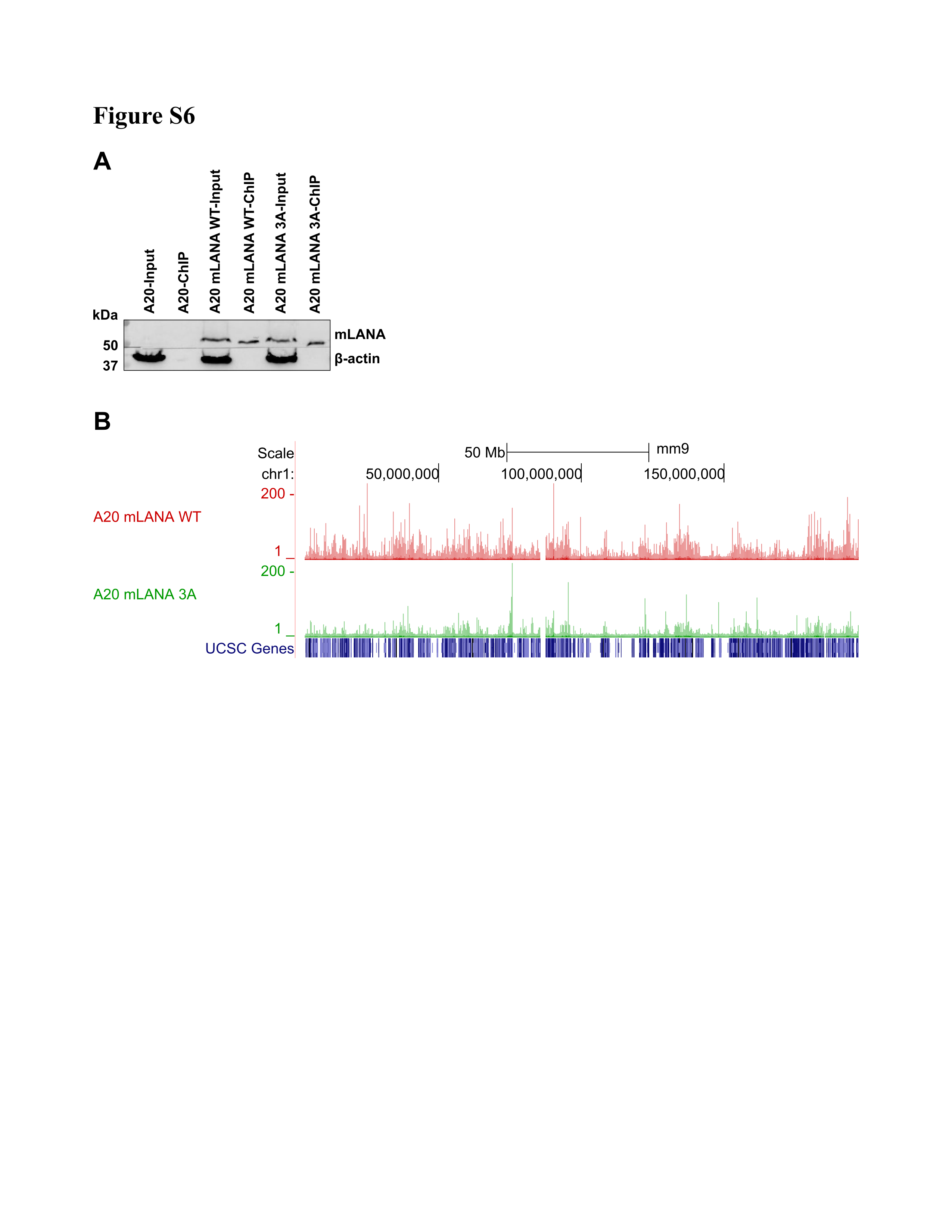

Supplement: Supplementary file 6 [file Image_6.TIFF]
