# Supplementary material for: Brd/BET Proteins Influence the Genome-Wide Localization of the Kaposi’s Sarcoma-Associated Herpesvirus and Murine Gammaherpesvirus Major Latency Proteins
Source: Front Microbiol. 2020 Oct 22;11:591778. doi: 10.3389/fmicb.2020.591778 (PMC7642799; doi:10.3389/fmicb.2020.591778)
Supplement: Supplementary file 7 [file Data_Sheet_1.docx]

**Supplementary Figures and Tables**

**Supplementary Figure S1**: **I-BET151 treatment induces low levels of lytic induction.** **(A)** Immunofluorescence assay for ORF59 and **(B)** quantification of number of ORF59-expressing cells as a percentage of all cells. Bars represent mean values (Mean ± SEM). **(C)** Western blot for kLANA and KbZIP.

**Supplementary Figure S2:** **Association of kLANA with the cellular genome as a function of Brd/BET proteins.** **(A)** Unique reads from ChIP-Seq experiments for kLANA, Brd2 and Brd4 (see Fig. 1) were aligned to the hg19 version of the human genome using Bowtie and peaks were detected using the MACS peak caller. The figure shows the distribution of ChIP-Seq reads on human chromosome 1 as a representative example. **(B)** kLANA, BRD2 and BRD4 are enriched in active chromatin regions of the human genome. Venn diagram for overlap of kLANA, BRD2, BRD4 and H3K4me3 (Hu et al., 2014) enriched regions on the host chromatin in BCBL-1 cells.

**Supplementary Figure S3: Motif analysis of kLANA enriched regions in BCBL-1 cells.** MEME-ChIP motif analysis for motif identification of kLANA enriched regions (top) and distribution of identified motif relative to position of best site in sequence by CentriMo analysis (bottom).

**Supplementary Figure S4: Expression of kLANA in BJAB-kLANA and BJAB-rKSHV.219 cells. (A)** Immunofluorescence for kLANA in virus negative BJAB-kLANA and virus positive BJAB-rKSHV.219 cells. Yellow box shows zoomed in view of a single cell to highlight the difference in kLANA staining between BJAB-kLANA and BJAB-rKSHV.219. BJAB-kLANA shows broad and diffuse kLANA staining whereas BJAB-rKSHV.219 cells show kLANA speckles typical of KSHV latency. **(B)** Western blot for kLANA in BJAB, BJAB-mCherry, BJAB-kLANA and BJAB-rKSHV219 cells.

**Supplementary Figure S5:** **Association of kLANA with cellular chromatin in the absence or presence of the viral genome. (A)** ChIP-Seq was performed in BJAB-kLANA and BJAB-rKSHV.219 cells following treatment with DMSO or 0.5 µM I-BET151 for 48 hours. Association of kLANA in BJAB-kLANA and BJAB-rKSHV.219 cells with the cellular genome. Unique reads were aligned to the hg19 version of the human genome. Peaks were detected using the MACS peak caller. The panel shows the distribution of ChIP-Seq reads on human chromosome 1 as a representative example. **(B)** MEME-ChIP motif analysis for the identification of a consensus binding motif in the cellular DNA of kLANA enriched regions (top left BJAB-kLANA and top right BJAB-rKSHV.219) and the distribution of an identified consensus motif relative to position of best site in sequence by CentriMo analysis (bottom).

**Supplementary Figure S6: Brd/BET-mediated chromatin association of mLANA. (A)** Western blot for mLANA in A20-GFP, A20 mLANA WT and A20 mLANA 3A cells. **(B)** ChIP-Seq was performed in A20-GFP, A20-mLANA WT and A20-mLANA 3A cells that were treated with doxycycline at a final concentration of 1 µg/ml for 48 hours. Association of MHV-68 mLANA WT and mLANA 3A mutant with the cellular genome. Unique reads were aligned to the mm9 version of the mouse genome. Peaks were detected using the MACS peak caller. Enrichment shown for mouse chromosome 1 as a representative.

**Supplementary Table ST1:** **kLANA-mediated cellular transcriptional regulation.** List of differentially regulated genes bound by kLANA at the TSS in the respective cell lines. Values in parentheses are Log_2_ fold change. Positive values denote upregulation and negative values denote downregulation. The gene names for ANO10, HECTD2 and DNMT3A are repeated due to their different transcript variants. Genes presented in bold have all three proteins (kLANA, BRD2, BRD4) bound at their TSS.

| **BJAB-kLANA**  **Upregulated** | **BJAB-kLANA**  **Downregulated** | **BJAB-rKSHV.219**  **Upregulated** | **BJAB-rKSHV.219**  **Downregulated** |
| --- | --- | --- | --- |
| ANO10 (0.63) | AFF2 (-0.61) | **DNMT3A (0.74)** | ACSS1 (-0.97) |
| ANO10 (0.70) | ARNTL2 (-0.61) | **DNMT3A (1.29)** | **FBXO4 (-1.03)** |
| DMKN (0.62) | ARSB (-0.63) | **NME6 (1.17)** |  |
| FCHO2 (0.77) | CTSZ (-1.03) | PTPDC1 (0.97) |  |
| FND3CA (0.84) | **HECTD2 (-1.43)** |  |  |
| MOB1B (0.59) | **HECTD2 (-1.56)** |  |  |
| SELPLG (0.63) | MCAT (-0.69) |  |  |
|  | MRPL38 (-0.68) |  |  |
|  | **NIPAL2 (-1.63)** |  |  |
|  | NT5DC3 (-0.71) |  |  |
|  | PAPSS2 (-1.21) |  |  |
|  | SLC22A5 (-0.76) |  |  |
|  | SLC39A8 (-0.96) |  |  |
